# MRI-guided histology of TDP-43 knock-in mice implicates parvalbumin interneuron loss, impaired neurogenesis and aberrant neurodevelopment in amyotrophic lateral sclerosis-frontotemporal dementia

**DOI:** 10.1093/braincomms/fcab114

**Published:** 2021-05-27

**Authors:** Ziqiang Lin, Eugene Kim, Mohi Ahmed, Gang Han, Camilla Simmons, Yushi Redhead, Jack Bartlett, Luis Emiliano Pena Altamira, Isobel Callaghan, Matthew A White, Nisha Singh, Stephen Sawiak, Tara Spires-Jones, Anthony C Vernon, Michael P Coleman, Jeremy Green, Christopher Henstridge, Jeffrey S Davies, Diana Cash, Jemeen Sreedharan

**Affiliations:** Department of Basic and Clinical Neuroscience, The Maurice Wohl Clinical Neuroscience Institute, Institute of Psychiatry, Psychology and Neuroscience (IoPPN), King’s College London, London SE5 9RT, UK; West China School of Medicine, West China Hospital, Sichuan University, Chengdu 610041, China; BRAIN Centre (Biomarker Research And Imaging for Neuroscience), Department of Neuroimaging, IoPPN, King’s College London, London SE5 9NU, UK; Centre for Craniofacial and Regenerative Biology, Floor 27 Tower Wing, Guy’s Hospital, King’s College London, London SE1 9RT, UK; Molecular Neurobiology Group, Institute of Life Sciences, School of Medicine, Swansea University, Swansea SA2 8PP, UK; BRAIN Centre (Biomarker Research And Imaging for Neuroscience), Department of Neuroimaging, IoPPN, King’s College London, London SE5 9NU, UK; Centre for Craniofacial and Regenerative Biology, Floor 27 Tower Wing, Guy’s Hospital, King’s College London, London SE1 9RT, UK; Molecular Neurobiology Group, Institute of Life Sciences, School of Medicine, Swansea University, Swansea SA2 8PP, UK; Department of Basic and Clinical Neuroscience, The Maurice Wohl Clinical Neuroscience Institute, Institute of Psychiatry, Psychology and Neuroscience (IoPPN), King’s College London, London SE5 9RT, UK; Department of Basic and Clinical Neuroscience, The Maurice Wohl Clinical Neuroscience Institute, Institute of Psychiatry, Psychology and Neuroscience (IoPPN), King’s College London, London SE5 9RT, UK; Department of Basic and Clinical Neuroscience, The Maurice Wohl Clinical Neuroscience Institute, Institute of Psychiatry, Psychology and Neuroscience (IoPPN), King’s College London, London SE5 9RT, UK; BRAIN Centre (Biomarker Research And Imaging for Neuroscience), Department of Neuroimaging, IoPPN, King’s College London, London SE5 9NU, UK; School of Biomedical Engineering & Imaging Sciences, St Thomas' Hospital, King's College London, 4th floor Lambeth Wing, London SE1 7EH, UK; Department of Clinical Neurosciences, Cambridge University, Cambridge CB2 0QQ, UK; Centre for Discovery Brain Sciences, University of Edinburgh, Edinburgh EH8 9XD, UK; Department of Basic and Clinical Neuroscience, The Maurice Wohl Clinical Neuroscience Institute, Institute of Psychiatry, Psychology and Neuroscience (IoPPN), King’s College London, London SE5 9RT, UK; Brain Repair Centre, Cambridge University, Cambridge CB2 0PY, UK; Centre for Craniofacial and Regenerative Biology, Floor 27 Tower Wing, Guy’s Hospital, King’s College London, London SE1 9RT, UK; Centre for Discovery Brain Sciences, University of Edinburgh, Edinburgh EH8 9XD, UK; Division of Systems Medicine, School of Medicine, University of Dundee, Dundee DD1 9SY, UK; Molecular Neurobiology Group, Institute of Life Sciences, School of Medicine, Swansea University, Swansea SA2 8PP, UK; BRAIN Centre (Biomarker Research And Imaging for Neuroscience), Department of Neuroimaging, IoPPN, King’s College London, London SE5 9NU, UK; Department of Basic and Clinical Neuroscience, The Maurice Wohl Clinical Neuroscience Institute, Institute of Psychiatry, Psychology and Neuroscience (IoPPN), King’s College London, London SE5 9RT, UK

**Keywords:** frontotemporal dementia, amyotrophic lateral sclerosis, TDP-43, parvalbumin, magnetic resonance imaging

## Abstract

Amyotrophic lateral sclerosis and frontotemporal dementia are overlapping diseases in which MRI reveals brain structural changes in advance of symptom onset. Recapitulating these changes in preclinical models would help to improve our understanding of the molecular causes underlying regionally selective brain atrophy in early disease. We therefore investigated the translational potential of the TDP-43^Q331K^ knock-in mouse model of amyotrophic lateral sclerosis-frontotemporal dementia using MRI. We performed *in vivo* MRI of TDP-43^Q331K^ knock-in mice. Regions of significant volume change were chosen for *post-mortem* brain tissue analyses. *Ex vivo* computed tomography was performed to investigate skull shape. Parvalbumin neuron density was quantified in post-mortem amyotrophic lateral sclerosis frontal cortex. Adult mutants demonstrated parenchymal volume reductions affecting the frontal lobe and entorhinal cortex in a manner reminiscent of amyotrophic lateral sclerosis-frontotemporal dementia. Subcortical, cerebellar and brain stem regions were also affected in line with observations in pre-symptomatic carriers of mutations in *C9orf72*, the commonest genetic cause of both amyotrophic lateral sclerosis and frontotemporal dementia. Volume loss was also observed in the dentate gyrus of the hippocampus, along with ventricular enlargement. Immunohistochemistry revealed reduced parvalbumin interneurons as a potential cellular correlate of MRI changes in mutant mice. By contrast, microglia was in a disease activated state even in the absence of brain volume loss. A reduction in immature neurons was found in the dentate gyrus, indicative of impaired adult neurogenesis, while a paucity of parvalbumin interneurons in P14 mutant mice suggests that TDP-43^Q331K^ disrupts neurodevelopment. Computerized tomography imaging showed altered skull morphology in mutants, further suggesting a role for TDP-43^Q331K^ in development. Finally, analysis of human post-mortem brains confirmed a paucity of parvalbumin interneurons in the prefrontal cortex in sporadic amyotrophic lateral sclerosis and amyotrophic lateral sclerosis linked to *C9orf72* mutations. Regional brain MRI changes seen in human amyotrophic lateral sclerosis-frontotemporal dementia are recapitulated in TDP-43^Q331K^ knock-in mice. By marrying *in vivo* imaging with targeted histology, we can unravel cellular and molecular processes underlying selective brain vulnerability in human disease. As well as helping to understand the earliest causes of disease, our MRI and histological markers will be valuable in assessing the efficacy of putative therapeutics in TDP-43^Q331K^ knock-in mice.

## Teaser

Lin, Kim and colleagues use MRI to appreciate changes in TDP-43^Q331K^ knock-in mice akin to those seen in presymptomatic human amyotrophic lateral sclerosis-frontotemporal dementia. A histological survey implicates parvalbumin interneuron loss, impaired neurogenesis, microglial activation and aberrant neurodevelopment in disease pathogenesis. They confirm reduced parvalbumin interneurons in human amyotrophic lateral sclerosis.

## Graphical Abstract

**Graphical Abstract:**
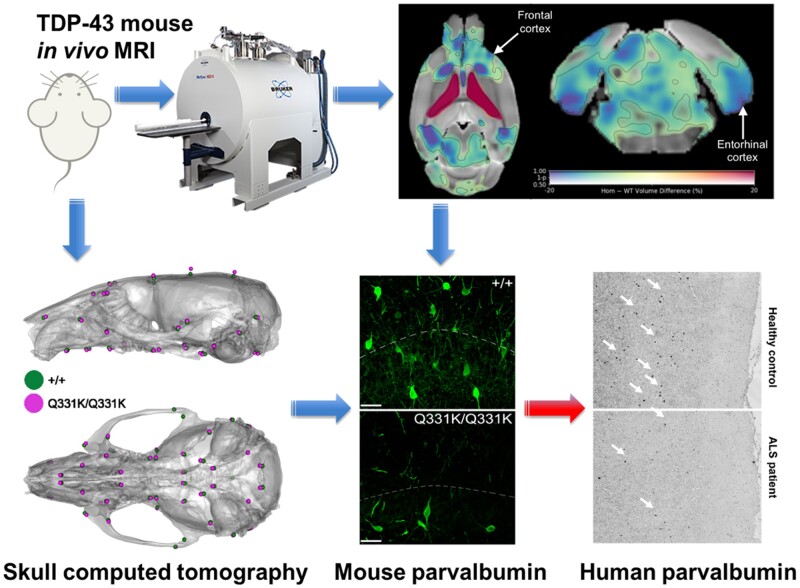

## Introduction

Amyotrophic lateral sclerosis (ALS) and frontotemporal dementia (FTD) exist on a clinicopathological and genetic disease spectrum (ALS-FTD). The loss of upper and lower motor neurons in ALS leads to paralysis, while FTD is characterized by frontal and temporal lobar degeneration causing executive dysfunction, language impairment and behavioural changes.^1^ From the point at which patients with ALS-FTD describe their first symptoms the decline is often rapid, with death ensuing within 3–5 years. However, it is increasingly apparent that patients can have seemingly asymptomatic prodromes that can extend for decades prior to clinical onset. This conclusion arises from studies using sensitive magnetic resonance imaging (MRI) methods, which have detected subtle deviations in brain structure or function in pre-symptomatic carriers of ALS-FTD-linked gene mutations.^2–5^ It follows that combining genetics with non-invasive imaging is a powerful approach to identify biomarkers of early disease.^6^ This is invaluable to enable early diagnosis and early therapeutic intervention to delay onset, slow progression or even prevent disease altogether, thereby providing the greatest possible benefit to patients.

Almost all patients with ALS and half of FTD cases are characterized by pathological processing of the RNA-binding protein TDP-43.^7–9^ Mutations in *TARDBP*, which encodes TDP-43, are also linked with ALS and FTD.^10^^,^^11^ These observations indicate key but undefined roles for TDP-43 in the pathogenesis of ALS-FTD. To elucidate molecular processes underlying the genesis of ALS-FTD it would be ideal to spatially resolve early brain changes *in vivo* and correlate regional atrophy with histological data in preclinical TDP-43 models. Such an approach promises to identify cross-species biomarkers of disease, with the potential to facilitate translational research. *In vivo* brain imaging, for example, offers the potential to directly correlate results from animal disease models with human imaging data. However, few such studies have been conducted in ALS-FTD models of disease, and these have not utilized an unbiased approach to identify affected brain regions of interest for further study.

Ideally, preclinical models should accurately reflect the human genetic state to ensure that observations are not due to artefacts caused by exogenous expression of transgenes. In the case of TDP-43, it is also important to ensure ubiquitous expression including throughout development.^12^ These criteria are fulfilled by the TDP-43^Q331K^ knock-in mouse, which harbours a single human-equivalent point mutation in the endogenous mouse *Tardbp* gene.^13^ We previously showed that this mutation causes a gain of function due to a loss of TDP-43 autoregulation, which leads to progressive behavioural features of FTD. Here, using *in vivo* MRI, *ex vivo* computed tomography (CT) and histology of TDP-43^Q331K^ knock-in mice, we make a significant translational step using this model. Firstly, we find a striking recapitulation of regional brain atrophy as seen in human ALS-FTD. We then use these MRI results to guide our histological studies, which implicate interneurons, microglia, impaired neurogenesis and aberrant neurodevelopment in early stage disease. Finally, we validate our interneuron observation using histology of human post-mortem brain tissues.

## Materials and methods

### Mouse breeding and maintenance

All animal experiments were performed under the UK Animal (Scientific Procedures) Act 1986 Amendment Regulations 2012 on Project Licences P023CC39A and P35785FD7. The creation of the TDP-43^Q331K^ knock-in mouse was previously described.^13^ For the current study, heterozygous (TDP-43^Q331K/+^) mice were intercrossed to generate TDP-43^Q331K/Q331K^, TDP-43^Q331K/+^ and wild-type offspring. All mice were bred on a C57Bl/6J background. Genotyping for the Q331K mutation was performed as described previously.^13^ Animals were group-housed in individually ventilated Tecniplast cages within a clean facility. Individual cages contained environmental enrichment items and group sizes of two to five mice were routinely maintained under a 12 h light/dark cycle with *ad libitum* access to food and water. Given that we previously found that males demonstrated more marked phenotypes than females,^13^ we focussed our attention on male mice in the current study.

### Structural magnetic resonance imaging

*In vivo* MRI was performed on 7-month-old (28–32 weeks, 27.3–38.6 g) male mice (10 TDP-43^Q331K/+^, 10 TDP-43^Q331K/Q331K^ and 15 wild-type littermates) using a 9.4 T horizontal bore Bruker BioSpec 94/20 scanner (Bruker BioSpin, Ettlingen, Germany). An 86-mm volume resonator and a 2 × 2 phased array surface coil were used for RF transmission and reception, respectively. During scanning, the mice were anaesthetized with 2–2.5% isoflurane. Their respiration rate and core temperature were monitored and maintained at 60–80 breaths/min and 37 ± 0.5°C (SA Instruments, Inc.). 3D T1-weighted images were acquired using an MPRAGE sequence with the following parameters: echo time = 3.8 ms, repetition time = 9.66 ms, inversion time = 1300 ms, flip angle = 10°, segment repetition time = 3500 ms, 4 segments, 4 averages, field of view = 16 mm × 16 mm × 8 mm, matrix = 128 × 128 × 64, scan time = 40 min. One wild-type and one TDP-43^Q331K/+^ mouse were excluded due to poor image quality.

Visual inspection of the MR images revealed that ventricular enlargement is a clear phenotype of homozygous TDP-43^Q331K/Q331K^ mutants. To quantify this using MATLAB (MathWorks, Natick, MA, USA) and FSL (FMRIB, Oxford, UK), the ventricles of each mouse were semi-automatically segmented through a combination of thresholding, morphological operations and manual editing if necessary. The volumes of these ventricle masks were compared across genotypes.

Tensor-based morphometry (TBM) was performed to assess the effects of mutant TDP-43^Q331K^ on local brain volume. First, N4 bias field correction was applied to the MPRAGE images to remove the signal inhomogeneity arising from the surface coil’s non-uniform sensitivity profile. Then, a study-specific template was created using ANTs (antsMultivariateTemplateConstruction2.sh). All subjects were registered to the template via sequential rigid, affine and non-linear SyN transforms (antsRegistration). The MPRAGE images and ventricle masks were given equal weight in the registration to improve the normalization of the largely varying ventricle sizes. Two TDP-43^Q331K/Q331K^ mice with frank hydrocephalus were excluded from further analysis because their ventricles could not be satisfactorily normalized to the template.

Jacobian determinant maps of the resultant deformation fields were computed and log-transformed. To compare local volume differences between mutant and wild-type mice, voxel-based nonparametric statistics were performed on the log-transformed Jacobian determinant maps using FSL randomize [5000 permutations, threshold-free cluster enhancement, family-wise error (FWE) correction]. An ANOVA was performed, followed by pair-wise comparisons between genotypes. We did not correct for global scaling as total brain volumes (i.e. brain plus ventricles) did not differ between genotypes.

The DSURQE mouse brain atlas (The Mouse Imaging Centre, Toronto)^14^ was similarly registered to the study-specific template for region of interest (ROI) analysis. For each mouse, ROI volumes were calculated by summing the Jacobian determinant values within each ROI. MATLAB was used to perform two-tailed Mann–Whitney U-tests to compare ROI volumes between each pair of groups, controlling for false discovery rate using the Benjamini–Hochberg step-up procedure.

### Mouse immunohistochemical studies

#### Tissue preparation

Following MRI, mice were culled by asphyxiation with CO_2_ followed by cervical dislocation and tissue extraction. The brains were immersion fixed in 4% paraformaldehyde at 4°C for 24–48 h, washed in phosphate buffered saline (PBS) and cryoprotected in 30% sucrose in PBS at 4°C for 72 h. The tissues were then embedded in M1 matrix in a silicon mould, frozen on dry ice, sectioned at 35 μm thickness on a cryostat (Leica) and stored in cyroprotectant (25% ethylene glycol, 30% glycerol) at −20°C. Tissue sections were then immunostained as follows (a list of the antibodies used is provided in Supplementary Table 1).

#### Immunostaining

For parvalbumin (PV) immunofluorescent staining cryoprotected sections were washed in PBS for 20 min, 0.2% TritonX-100/PBS for 10 min and blocked in 5% normal goat serum (NGS)/0.2% TritonX-100/PBS for 1 h at room temperature (RT). Sections were incubated overnight at 4°C with primary anti-PV antibody diluted in blocking buffer, washed in 0.2% TritonX-100/PBS, then incubated for 2 h at RT with secondary antibody diluted in blocking buffer, then washed in 0.2% TritonX-100/PBS. Sections were subsequently washed in PBS and mounted onto Superfrost Plus slides (VWR, France) with Vectashield HardSet with 4′,6-diamidino-2-phenylindole (DAPI) (Vector Laboratories, USA).

We assessed microglia by staining for ionized calcium-binding adapter molecule 1 (Iba-1), which is elevated in activated microglia, and transmembrane protein 119 (Tmem119). Tmem119 is poorly understood but is expressed on the cell surface of brain resident microglia, but not blood-derived macrophages^15^ and has roles in myoblast differentiation. Although its functions in microglia remain unclear the expression of Tmem119 protein has been shown to be reduced in disease-associated microglia (DAM).^16–18^ For Iba-1 and Tmem119 co-immunofluorescent staining, antigen retrieval was first performed by heating the samples for 20 min at 80°C in sodium citrate buffer (pH 6.5). Sections were cooled to RT, washed in distilled water, and blocked and permeabilized in a solution containing 5% bovine serum albumin, 0.5% Triton X-100 for 1 h at RT. Slides were incubated with primary antibody (goat anti-Iba1, Abcam, ab5076, 1:500) for 4°C overnight in 2-fold-diluted blocking buffer. Secondary antibody (donkey anti-goat, Alexa Fluor 488, 1:500) was applied for 1 h at RT. Sections were then washed and blocked again as above and incubated with primary antibody (rabbit anti-Tmem119, Abcam, ab209064, 1:1000) for 4°C overnight. Secondary antibodies were applied for 1 h at RT (goat anti-rabbit, Alexa Fluor 568, 1:500). Sections were counterstained and mounted with Vectashield HardSet with DAPI (Vector Laboratories, USA).

For DAB (3,3′-Diaminobenzidine) immunohistochemistry, sections were washed in 0.1 M phosphate buffered saline (PBS) and 0.1 M PBS-Tween (PBS-T). Endogenous peroxidases were quenched by washing in PBS plus 1.5% H_2_O_2_ for 20 min. Sections were washed again. Antigen retrieval was performed where needed (i.e. for myelin basic protein and Ki67 staining) in sodium citrate at 70°C for 1 h. Sections were blocked in 5% normal goat serum in PBS-T for 1 h, washed and then incubated overnight at 4°C with primary antibody in PBS-T and 2% normal goat serum solution. Sections were washed again prior to incubation with biotinylated secondary antibody in PBS-T for 70 min. Sections were washed and incubated in ABC solution (Vectastain ABC Elite, PK-6000, Vector Laboratories, USA) for 90 min in the dark, then washed twice more in PBS, and incubated with 0.1 M sodium acetate pH 6 for 10 min. Immunoreactivity was developed in DAB solution followed by two washes in PBS. Sections were mounted and allowed to dry overnight before being dehydrated and de-lipidized in increasing concentrations of ethanol. Sections were incubated in Histoclear (2 × 3 min; National Diagnostics, USA) and cover-slipped with mounting medium (Sigma Aldrich, UK).

#### Image analyses

For adult mouse immunostaining, images were captured using an Olympus Whole Slide Scanner (VS120, Olympus, Japan) with a ×20 objective. Z-stacks of 5 layers were obtained at ×20 magnification throughout each section. Images were auto-stitched using Olyvia software (Olympus). Images were analysed using Visiopharm image analysis software (Hoersholm, Denmark) blind to genotype. Anatomically equivalent sections were selected from 4 to 6 mice of each genotype. ROIs were manually drawn in each chosen section according to the Allen mouse brain atlas (Allen Institute for Brain Science). Each ROI extended through 3–6 sections depending on its size. Each marker was measured using a custom Visiopharm application via threshold classification and post processing changes. The number of any particular cellular subtype per tissue area (‘cell density’), and the proportion of an area positive for any marker (‘percentage area’), were calculated. The measurements in all sections for each ROI were summed for each mouse (density = total cell number/total tissue area; percentage area = total marker-positive area/total tissue area).

For quantification of PV+ interneurons in P14 mouse hippocampi, cells were manually quantified (30 µm confocal z-stacks, Leica TCS SP5) from six anatomically equivalent sections per animal within a given region using Fiji (ImageJ). Watershed algorithm was used to define the ROI and DAPI+ cells quantified with the parameters—radius: 0.5; min level: 90–127; max level: 127–192; erode/dilate iterations: 0 in binary images. Mean pixel intensities were checked for accuracy by redirecting measurements to the original image for overlay with DAPI. The fold change of immunostaining in all animals for each ROI was calculated relative to the mean value of wild-type controls.

### *Ex vivo* micro-CT imaging and data analysis

For micro-CT, 24-month-old male mice were perfused with 2% paraformaldehyde, and heads were processed as previously described.^19^ Heads were scanned using a Scanco µCT50 micro-CT scanner (Scanco, Brüttisellen, Switzerland). The specimens were immobilized in 19 mm scanning tubes using cotton gauze and scanned to produce 20 µm voxel size volumes, using X-ray settings of 70 kVp, 114 µA and a 0.5 mm aluminium filter to attenuate harder X-rays. The scans were automatically scaled at reconstruction using the calibration object provided by the CT manufacturer, consisting of five rods of hydroxyapatite at concentrations of 0 to 790 mg/cm^3^, and the absorption values expressed in Hounsfield units.

For CT image analysis, 53 landmarks of the skull were obtained by reconstructing a 3D surface of the micro-CT images using Microview software by Parallax Innovations (http://www.parallax-innovations.com/microview.html, last accessed 7 June 2021) followed by genotype-blinded manual placement and orthogonal-view cross-checking. The MorphoJ software package^20^ was used to apply Procrustes superimposition to landmarks for alignment after which shape variation was visualized through principal component analysis. Procrustes Distance Multiple Permutations test (1000 iterations) was used to quantify the significance of shape differences between genotypes. Size differences were compared by measuring the centroid size (square root of the sum of the squared distances from each landmark to the centroid) and significance measured via an unpaired *t*-test. Videos were generated by taking one representative homozygous mutant and one representative wild-type mouse and using a pipeline as previously described^21^ to morph between them.

### Human tissue studies

#### Patient details

All patients had clinical and electrophysiological evidence of combined upper and lower motor neuron dysfunction and fulfilled the revised El Escorial criteria for a diagnosis of ALS.^22^ Patients were recruited through the Scottish Motor Neurone Disease Register and data collected in the CARE-MND database (Clinical Audit Research and Evaluation—motor neurone disease). Ethical approval for this register was obtained from Scotland A Research Ethics Committee 10/MRE00/78 and 15/SS/0216. All clinical data were subsequently extracted from the CARE-MND database.

Patients gave pre-mortem consent for brain and spinal cord donation. All tissue retrievals were in line with the Human Tissue (Scotland) Act, and all procedures have been approved by a national ethics committee. Use of human tissue for post-mortem studies has been reviewed and approved by the Sudden Death Brain Bank ethics committee and the ACCORD medical research ethics committee, AMREC (ACCORD is the Academic and Clinical Central Office for Research and Development, a joint office of the University of Edinburgh and NHS Lothian). Details of all donors used in this study (ages, sex and *C9orf72* mutation status) with means for each group can be found in Supplementary Tables 2 and 3.

#### Neuropathology

Fresh post-mortem tissue blocks of human brain were fixed in 10% formalin for a minimum of 24 h. Tissue was dehydrated in an ascending alcohol series (70–100%), followed by three xylene washes, all for 4 h each. Next, three paraffin waxing stages (5 h each) were performed to ensure full penetration of the embedding wax and the blocks were cooled. Tissue sections were cut on a Leica microtome at 4 μm and collected on glass slides. All sections were dried at 40°C for at least 24 h before staining. Immunohistochemistry was performed using standard protocols, enhanced using a sensitive avidin/biotin kit (VECTASTAIN ABC Elite) and visualized using DAB as chromogen. The primary antibody was rabbit anti-Parvalbumin (Swant, PV27, 1:1000). Slides were finally counterstained with haematoxylin for 30 s to stain cell nuclei. All staining was performed by investigators blind to clinical diagnosis.

One section per case was analysed and PV+ cell densities were generated using Stereo Investigator. Cortical grey matter was outlined in each section and immune-positive cells identified using an automated colour-based thresholding algorithm. The accuracy of this automated process was assessed on each section by manually checking the identified objects. The number of immuno-positive cells was expressed as a cell density by dividing the total number by the total cortex area, expressed as PV+ cells/mm^2^. All imaging and analysis were performed by investigators blind to clinical diagnosis.

### Experimental design and statistical analysis

Experimental data were analysed with the operator blind to the genotype of animals. All statistical analyses were conducted, and all figures plotted using Prism 8.3.0 (GraphPad Software, Inc.). All statistical comparisons are based on biological replicates unless stated otherwise. All charts show mean ± SEM and statistical tests used are described in the relevant results or figure legends. *P*-values <0.05 were considered significant for all statistical analyses used unless otherwise indicated.

### Data availability

The datasets generated and/or analysed during the current study are available in the OpenNeuro repository, https://openneuro.org/datasets/ds003325/versions/1.0.0, last accessed 7 June 2021.

## Results

### Regional parenchymal volume loss and ventricular enlargement on *in vivo* MRI

**Figure 1:**
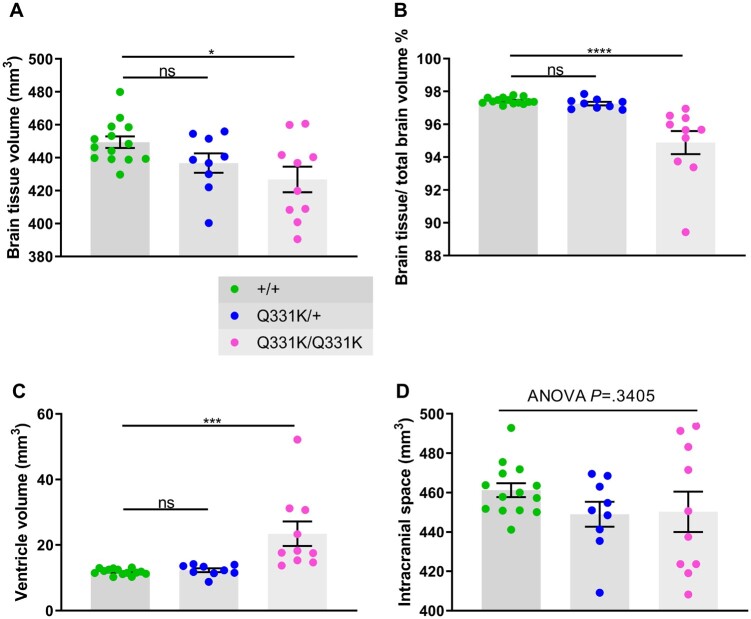
*In vivo* MRI reveals global brain volume loss and ventricular enlargement in 7-month-old TDP-43^Q331K/Q331K^ knock-in mice. (A) Quantification of brain parenchyma volume. ANOVA *P* = 0.0191. Pairwise comparisons: +/+ versus Q331K/+: ns *P* = 0.1159; +/+ versus Q331K/Q331K: * *P* = 0.0115. (**B**) Quantification of brain parenchyma volume as a percentage of total brain volume. ANOVA *P* < 0.0001. Pairwise comparisons: +/+ versus Q331K/+: ns *P* = 0.7448; +/+ versus Q331K/Q331K: **** *P* < 0.0001. (**C**) Quantification of ventricle volume. ANOVA *P* = 0.0003. Pairwise comparisons: WT (+/+) and TDP43^Q331K/+^ (Q331K/+): ns *P* = 0.8597; WT (+/+) and TDP43^Q331K/Q331K^ (Q331K/Q331K): *** *P* = 0.0004. (**D**) Quantification of brain parenchyma volume and ventricular volume combined. ANOVA *P* = 0.3405. (**A–D**) Each dot represents one mouse. Groups were compared by one-way ANOVA followed by Holm**–**Sidak *post hoc* test for pairwise comparisons; error bars represent mean ± SEM.

**Figure 2:**
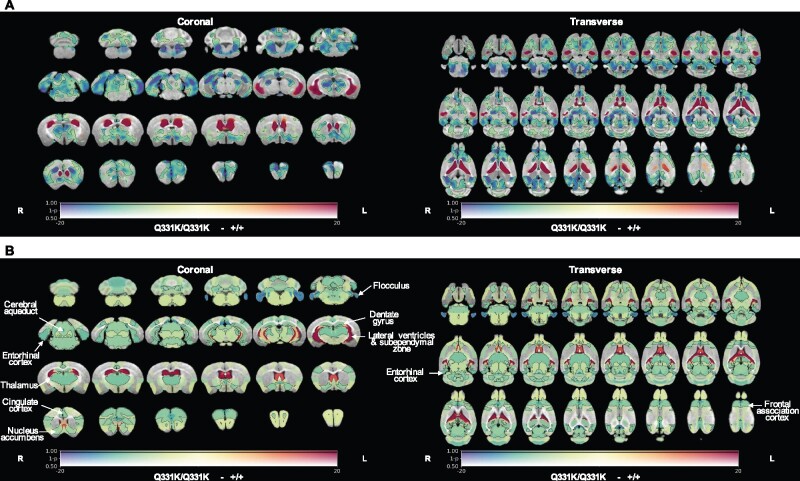
***In vivo* MRI reveals regional brain volume loss and ventricular enlargement in 7-month-old TDP-43^Q331K/Q331K^ knock-in mice.** (A) A map of voxel-wise differences in volume between 7-month-old +/+ and Q331K/Q331K mice calculated from *in vivo* MR images, overlaid on the T1-weighted study-specific template. The map is displayed in the coronal plane (rostral-caudal) and the transverse plane (ventral–dorsal). The R and L indicate the right and left sides of the mouse. The colours of the overlay indicate the percent volume difference (cool colours indicate reduced volume in Q331K/Q331K mice compared to +/+ mice), and the opacity of the overlay indicates the significance of the volume difference (regions where the FWE-corrected *P* > 0.5 are completely transparent, and regions where the FWE-corrected *P* = 0 completely opaque). Black contours demarcate regions where the FWE-corrected *P* < 0.05. (**B**) A similar map as in (**A**) but showing differences in the DSURQE atlas ROI volumes. Black contours demarcate ROIs where the false discovery rate-corrected *P* < 0.05. ROIs of particular interest are annotated.

To identify structural brain changes caused by mutant TDP-43^Q331K^ we performed *in vivo* MRI on 7-month-old male heterozygous (TDP-43^Q331K/+^) and homozygous (TDP-43^Q331K/Q331K^) mutant mice and wild-type littermates. This represents an early symptomatic stage in this model with evidence of executive dysfunction, but no motor neuron loss.^13^ MRI revealed that the brain parenchymal volume decreased by 5.0% (Fig. 1A and B), with homozygous mutants demonstrating a range of volumetric changes, indicating phenotypic heterogeneity as previously described.^13^ Furthermore, there was a striking 49.7% increase in total ventricular volume in TDP-43^Q331K/Q331K^ mice compared to wild-types (Fig. 1C). There was no significant difference in total brain and ventricular volume combined (Fig. 1D). Furthermore, voxelwise analysis using TBM^19^ revealed widespread statistically significant (FWE *P *<* *0.05) volume reductions in cortical, subcortical and cerebellar regions in TDP-43^Q331K/Q331K^ compared to wild-type mice (Fig. 2A).

To more precisely define patterns of atrophy and identify brain areas most vulnerable to mutant TDP-43^Q331K^, we conducted an ROI analysis using the DSURQE mouse brain atlas. This allowed us to compare the volumes of 182 pre-defined regions between the groups of mice. Fifty-one regions with statistically significant volume changes between TDP-43^Q331K/Q331K^ and wild-type mice were identified after correction for multiple comparisons at 5% false discovery rate (Table 1 and Fig. 2B). The most significant cortical volume reductions occurred in the frontal, motor, entorhinal, orbital and cingulate cortices, while subcortical volume loss was most marked in the nucleus accumbens, thalamus, subiculum, hippocampus, basal forebrain and the brain stem. In the cerebellum, there were decreases in the flocculus and the deep nuclei (dentate and interpositus), as well as in the cerebro-cerebellar white matter tracts (cerebellar peduncles). The most significant volume increase was, as expected, in the lateral ventricles, although the volumes of the third and fourth ventricles were not significantly affected while the cerebral aqueduct was decreased in size (Table 1). We also detected apparent volume increases in several grey and white matter ROIs, notably the CA3 hippocampal field and fimbria. However, these are likely to be artefacts caused by the proximity of these ROIs to the enlarged lateral ventricles, which can cause signal contamination due to partial volume effects, imperfect normalization and smoothing used in tensor-based morphometry analysis.^25^ This is illustrated in Supplementary Fig. 1, which shows the ventricles of the normalized subjects extending slightly beyond the ventricle ROIs of the atlas; the image registration was otherwise very good. Overall, the pattern of regional brain volume reduction in TDP-43^Q331K/Q331K^ mice was reminiscent of that seen in patients with ALS-FTD and in presymptomatic carriers of ALS-FTD-linked gene mutations (Table 1).

**Table 1:** Atlas ROIs with significantly altered volumes in TDP-43^Q331K/Q331K^ mice

Brain area	Affected brain region (DSURQE mouse brain atlas)^23^	Difference in median volume (mm^3^)/% (false discovery rate adj. *P-*value)	Equivalent human brain region	Human ALS and FTD imaging evidence
Cortex and hippocampus	Accessory olfactory bulb: glomerular, external plexiform and mitral cell layer	−0.05/−9.28 (0.03)	Olfactory bulb (frontal lobe)	No evidence in FTD, only in early AD^24^
	Dorsal taenia tecta	−0.10/−8.52 (0.03)		
	Accessory olfactory bulb: granule cell layer	−0.02/−7.24 (0.03)		
	Frontal association cortex	−0.79/−9.83 (0.03)	Prefrontal cortex/Frontal lobe	^2^ ^,^ ^25–36^ Presymptomatic:^4^^,^^5^^,^^37–39^
	Frontal cortex: area 3	−0.05/−6.61 (0.03)		
	Cingulate cortex: area 32	−0.25/−9.47 (0.03)	Cingulate cortex	^2^ ^,^ ^26^ ^,^ ^27^ ^,^ ^36^ Presymptomatic:^37–39^
	Cingulate cortex: area 24a'	−0.07/−9.59 (0.05)		
	Cingulate cortex: area 29a	−0.12/−15.98 (0.03)		
	Lateral orbital cortex	−0.30/−9.17 (0.03)	Orbitofrontal cortex	^2^ ^,^ ^25^ ^,^ ^26^ ^,^ ^30^ ^,^ ^32^ ^,^ ^40^ Presymptomatic:^2^^,^^41^
	Medial orbital cortex	−0.26/−13.06 (0.03)		
	Primary somatosensory cortex	−0.40/−9.74 (0.04)	Somatosensory cortex/parietal lobe	^2^ ^,^ ^26^ ^,^ ^28^ ^,^ ^30^ ^,^ ^35^ Presymptomatic:^2^^,^^4^^,^^5^^,^^37^^,^^39^^,^^42^
	Caudomedial entorhinal cortex	−0.68/−11.48 (0.02)	Anterior and medial temporal lobe	^2^ ^,^ ^25^ ^,^ ^28–30^ ^,^ ^40^ ^,^ ^43–45^ Presymptomatic:^2^^,^^4^^,^^5^^,^^37^^,^^39^^,^^41^^,^^42^
	Medial entorhinal cortex	−0.07/−10.63 (0.02)		
	Ventral intermediate entorhinal cortex	−0.11/−8.99 (0.03)		
	CA30r	0.63/20.66 (0.03)	Hippocampus/temporal lobe	^2^ ^,^ ^26^ ^,^ ^31^ ^,^ ^35^ ^,^ ^36^ ^,^ ^43^ ^,^ ^44^ ^,^ ^46^ Presymptomatic^4^^,^^37^^,^^47^
	CA3Py Outer	0.15/16.51 (0.03)		
	Pre-para subiculum	−0.22/−9.54 (0.03)		
	Subiculum	−0.33/−10.29 (0.03)		
	MoDG (dentate gyrus)	−0.40/−10.03 (0.03)		
	SLu	0.06/10.71 (0.05)		
	Primary visual cortex: binocular area	−0.21/−10.17 (0.03)	Occipital/posterior cortex	^28^ ^,^ ^30^ Presymptomatic:^37^
	Intermediate nucleus of the endopiriform claustrum	−0.04/−6.99 (0.04)	Piriform cortex	
Subcortical grey matter	Fundus of striatum	−0.01/−7.92 (0.03)	Basal ganglia	^2^ ^,^ ^25^ ^,^ ^28^ ^,^ ^32^ ^,^ ^34^ ^,^ ^36^ ^,^ ^45^ ^,^ ^48^ Presymptomatic:^47^
	Lateral septum	0.48/15.00 (0.03)	Lateral septum	
	Claustrum	−0.03/−11.92 (0.03)	Claustrum/insula	
	Nucleus accumbens	−0.34/−7.97 (0.03)	Nucleus accumbens/ventral striatum	^25^ ^,^ ^26^ ^,^ ^32^ ^,^ ^45^ ^,^ ^48^ Presymptomatic:^47^
	Thalamus	−1.98/−10.57 (0.03)	Thalamus	^2^ ^,^ ^25^ ^,^ ^26^ ^,^ ^28^ ^,^ ^33^ ^,^ ^35^ ^,^ ^36^ ^,^ ^38^ ^,^ ^49^ ^,^ ^50^ Presymptomatic:^2^^,^^4^^,^^5^^,^^37^^,^^39^^,^^42^^,^^47^
	Basal forebrain	−0.46/−9.59 (0.05)	Basal forebrain	Not reported. Only in MCI^51^
Mid-brain	Colliculus: superior	−0.82/−9.03 (0.03)	Superior colliculus	^33^
	Periaqueductal grey	−0.30/−8.54 (0.03)	Periaqueductal grey	^27^
	Colliculus: inferior	−0.64/−10.29 (0.03)	Inferior colliculus	^33^
Brain stem	Medulla	−1.59/−5.17 (0.03)	Medulla	^25^ ^,^ ^26^ ^,^ ^33^
	Pons	−1.68/−9.26 (0.05)	Pons	
Cerebellum	Flocculus (FL)	−0.10/−12.14 (0.03)	Flocculus	^2^ ^,^ ^25^ ^,^ ^26^ ^,^ ^28^ ^,^ ^30^ ^,^ ^52^ ^,^ ^53^ Pre-symptomatic:^2^^,^^4^^,^^37^^,^^39^^,^^42^
	Nucleus interpositus	−0.05/−11.83 (0.03)	Nucleus interpositus	
	Dentate nucleus	−0.02/−8.81 (0.04)	Dentate nucleus	
Ventricular system	Lateral ventricle	2.08/57.43 (0.01)	Lateral ventricle	^25^ ^,^ ^31^ ^,^ ^46^ ^,^ ^50^ Presymptomatic:^47^^,^^54^
	Cerebral aqueduct	−0.05/−15.57 (0.02)	Cerebral aqueduct	
	Subependymal zone/rhinocele	−0.005/−9.04 (0.04)	Subependymal zone	
Cerebral white matter	Fimbria	0.74/23.50 (0.01)	Fimbria	
	Anterior commissure: pars posterior	−0.04/−9.16 (0.03)	Anterior commissure: pars posterior	
	Fasciculus retroflexus	−0.01/−6.87 (0.03)	Fasciculus retroflexus	
	Stria medullaris	−0.05/−7.22 (0.03)	Stria medullaris	
	Anterior commissure: pars anterior	−0.13/−10.49 (0.04)	Anterior commissure: pars anterior	
	Mammilothalamic tract	−0.02/−11.03 (0.04)	Mammilothalamic tract	
Cerebellar white matter	Flocculus white matter	−0.004/−10.37 (0.03)	Flocculus white matter	^26^
	Cerebellar peduncle: inferior	−0.11/−14.66 (0.03)	Cerebellar peduncles	
	Cerebellar peduncle: superior	−0.12/−9.18 (0.03)		
	Paraflocculus white matter	−0.03/−13.44 (0.04)	Paraflocculus white matter	
	Trunk of arbour vita	−0.47/−9.71 (0.05)	Trunk of arbour vita	

Structures with median volume (mm^3^) and % difference between TDP43^Q331K/Q331K^ and wildtype mice that are significant at *P* < 0.05 after false discovery rate correction, and the corresponding or equivalent human brain areas as well evidence of similar/related changes reported in human ALS and FTD imaging literature. Pre-symptomatic: asymptomatic carriers of mutations in C9orf72, MAPT or GRN.

Comparison of heterozygous TDP-43^Q331K/+^ with wild-type mice yielded no statistically significant volume differences after correction for multiple comparisons at 5% false discovery rate. However, TDP-43^Q331K/+^ mice did demonstrate trends towards volume loss or gain in several brain regions that were significant in TDP-43^Q331K/Q331K^ mice (see Supplementary Fig. 2). This suggests a dose-effect of the mutation on brain structural phenotypes. Indeed, we made analogous observations in our previous study with TDP-43^Q331K/+^ mice, which demonstrated similar behavioural and transcriptomic changes to those seen in TDP-43^Q331K/Q331K^ mice, but of smaller magnitude.^13^ We therefore focused our attention on comparing wild-type with TDP-43^Q331K/Q331K^ mice in subsequent histological analyses in order to identify cellular correlates of volume loss on MRI.

### Reduced parvalbumin interneuron density in ROIs

**Figure 3:**
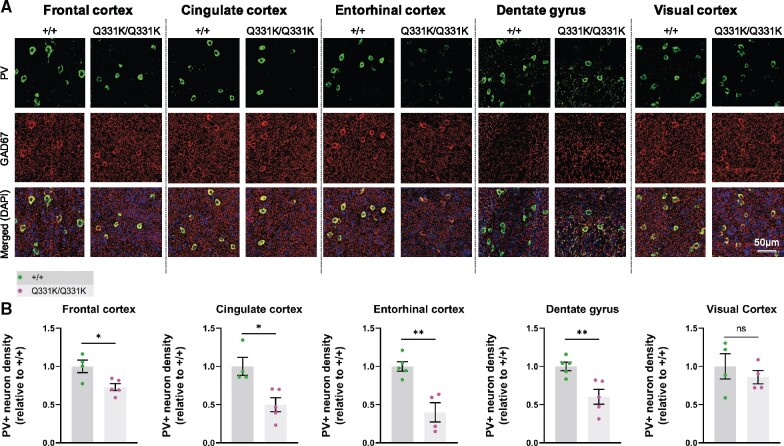
**Parvalbumin neuron density is reduced in MRI regions of interest in 7-month-old TDP-43^Q331K/Q331K^ mice.** (A) Representative images showing parvalbumin (PV) staining, GAD67 staining, and DAPI (blue) in indicated regions of 7-month-old WT (+/+) and TDP43^Q331K/Q331K^ (Q331K/Q331K) mouse brains. (**B**) Quantification of PV^+^ interneuron density in given brain regions. Each dot represents one mouse. Comparisons between WT and TDP43^Q331K/Q331K^ as follows: frontal cortex * *P* = 0.0186; cingulate cortex, * *P* = 0.0107; entorhinal cortex, ** *P* = 0.0026; dentate gyrus, ** *P* = 0.0074; visual cortex, *P* = 0.4742 (ns, not significant). *P*-values were calculated with multiple *t*-tests adjusted by Holm–Sidak correction. Error bars represent mean ± SEM.

We previously observed a reduced density of PV+ interneurons as well as reduced PV mRNA expression at both 5 and 20 months of age in frontal cortices of TDP-43^Q331K/Q331K^ mice.^13^ Given that reduced PV+ neuron density and a loss of other gamma-aminobutyric acid (GABA)ergic inhibitory interneuron subtypes is also observed in patients with ALS,^55–57^ we reasoned that interneuron readouts could be a sensitive indicator of regional brain vulnerability in ALS-FTD. We therefore immunostained for PV and the GABA synthetic enzyme glutamic acid decarboxylase 1 (GAD1/GAD67) in four ROIs demonstrating volume loss on MRI. We also analysed PV+ interneuron density in the visual cortex as a negative control as this area was largely unaffected on MRI (Fig. 3A and B). We found that the frontal cortex showed a reduction (by 27.1%) in PV+ interneuron density in mutant mice. The cingulate cortex was one of the most significantly affected subregions within the frontal cortex, demonstrating a 50.1% reduction of PV+ interneuron density. The entorhinal cortex and the dentate gyrus of the hippocampus demonstrated 60.2% and 39.8% reductions in PV+ interneuron density, respectively. By contrast, there was no significant reduction in PV+ interneuron density in the visual cortex. Finally, to determine if other interneuron subtypes could be affected by mutant TDP-43^Q331K^ we quantified somatostatin-positive (SOM+) interneurons. No significant change in SOM+ interneuron density was seen in mutant mice (see Supplementary Fig. 3A and B). We also examined overall GAD expression but saw no significant change in GAD expression in mutant mice (Supplementary Fig. 3C). Collectively, these results indicate that PV+ interneurons are selectively reduced in number in MRI regions that are significantly reduced in volume, and this may correlate with regions of volume loss on MRI.

### Widespread microglial activation

**Figure 4:**
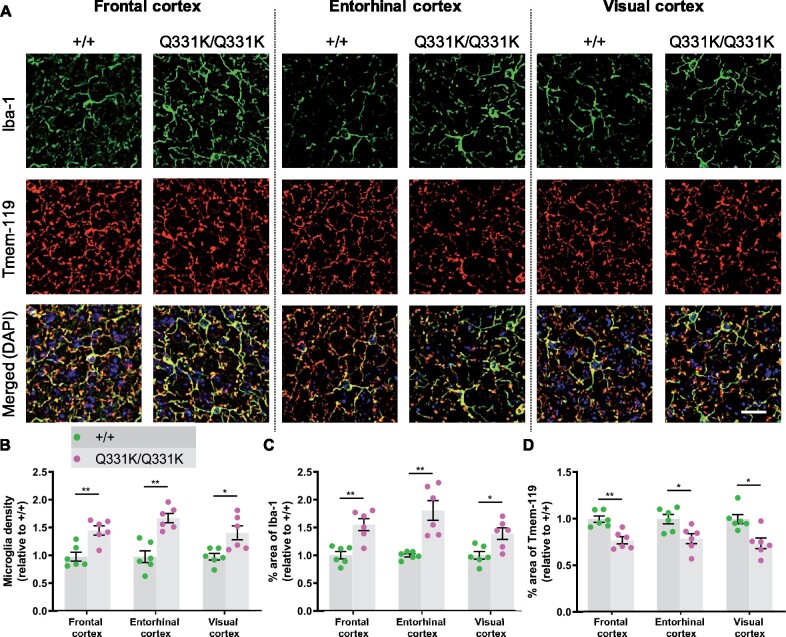
**Global microglial activation in the TDP-43^Q331K/Q331K^ mutant mouse brain.** (A) Immunohistochemistry showing microglia marker Iba-1 (green), Tmem119 (red) and DAPI (blue) in indicated regions of 7-month-old +/+ and Q331K/Q331K mouse brains. Scale bar, 20 μm. (**B**) Quantification of microglia density based on Iba-1 and DAPI immunoreactivity in given brain regions. Comparisons between +/+ and Q331K/Q331K as follows: frontal cortex, ** *P* = 0.0028; entorhinal cortex, ** *P* = 0.0012; visual cortex, * *P* = 0.0113. (**C**) Quantification of percentage area of Iba-1 in given brain regions. Comparisons between +/+ and Q331K/Q331K: frontal cortex, ** *P* = 0.0043; entorhinal cortex, ** *P* = 0.0035; visual cortex, * *P* = 0.0107. (**D**) Quantification of percentage area of Tmem119 in given brain regions. Comparisons between +/+ and Q331K/Q331K: frontal cortex, ** *P* = 0.0039; entorhinal cortex, * *P* = 0.0174; visual cortex, * *P* = 0.0122. (**B–D**) Each dot represents one mouse. *P*-values were calculated with multiple *t*-tests adjusted by Holm–Sidak correction. Error bars represent mean ± SEM.

Glial involvement in the pathogenesis of ALS-FTD is well established, with both astrocytic and microglial activation reported, while the presence of TDP-43 pathology in white matter implicates oligodendrocytes as well.^58–60^ To determine the responses of glia to mutant TDP-43^Q331K^, we used immunohistochemistry to study two MRI-defined brain ROIs, and again used the visual cortex as a negative control region. Iba1 immunostaining revealed significant increases in the area of staining and in microglial density in the frontal and entorhinal cortices of TDP-43^Q331K/Q331K^ mice, while Tmem119 immunostaining was significantly reduced in these regions (Fig. 4A–D). Surprisingly, the visual cortex also displayed both increased Iba1 and decreased Tmem119 staining. Astrocytes, however, demonstrated no evidence of activation or proliferation in mutant mice by immunostaining for glial fibrillary acidic protein (see Supplementary Fig. 4A and B). Immunostaining for myelin basic protein also showed no significant difference between mutant and wild-type mice, suggesting no change in myelination due to mutant TDP-43^Q331K^ (see Supplementary Fig. 4C and D). Collectively, these results indicate that the earliest glial change in TDP-43^Q331K^ knock-in mice is the transformation of microglia into a disease-associated state, and that this occurs even in the absence of volume loss on MRI.

### Reduced hippocampal neurogenesis

**Figure 5:**
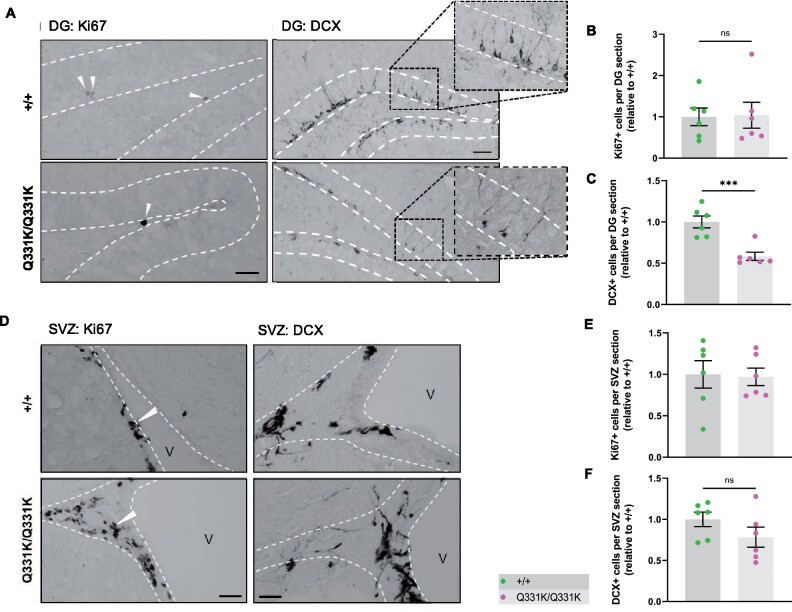
**Aberrant neurogenesis in TDP-43^Q331K/Q331K^ mice.** (A) Immunohistochemistry showing cell division marker Ki67 (arrow heads) and immature neuron marker DCX in the mouse hippocampal dentate gyrus of 7-month-old +/+ and Q331K/Q331K mice. Scale bar, 40 and 100 μm, representatively. (**B**) Quantification of Ki67+ cells per dental gyrus (DG) section in hippocampus between +/+ and Q331K/Q331K mice: ns *P* = 0.9183. (**C**) Quantification of DCX+ cells per DG section in the hippocampal dentate gyrus between +/+ and Q331K/Q331K mice: *** *P* = 0.0008. (**D**) Representative images showing cell division marker Ki67 (arrow heads) and immature neuron marker DCX in the mouse subventricular zone with (**E, F**) quantification. Comparisons between +/+ and Q331K/Q331K as follows: Ki67, ns *P* = 0.8820; DCX, ns *P* = 0.1794. V = lateral ventricle. (**B, C, E, F**) Each dot represents one mouse. Groups were compared by unpaired two-tailed *t*-test. All data shown are mean ± SEM.

Another region demonstrating significant volume loss on MRI and reduced PV+ interneuron density was the hippocampal dentate gyrus (DG). Given that this structure is an important niche for adult hippocampal neurogenesis (AHN) and that PV+ interneurons have an essential role in neurogenesis,^61^^,^^62^ we quantified markers of cell proliferation (Ki67) and immature neuron formation [doublecortin (DCX)] in the granule cell layer of the DG. We found no difference in the number of Ki67+ cells between wild-type and TDP-43^Q331K/Q331K^ mice, suggesting that the rate of progenitor cell division was unaffected (Fig. 5A and B). However, quantification of DCX+ cells revealed a significant reduction in the number of immature neurons in the granule cell layer of TDP-43^Q331K/Q331K^ mice (Fig. 5A and C). Given the volume changes in the lateral ventricles we also examined the subventricular zone, another key area of adult neurogenesis, but found no evidence of impaired neurogenesis here (Fig. 5D–F). Collectively, these results indicate that mutant TDP-43^Q331K^ reduces the number of progenitor cells undergoing neuronal differentiation or, alternatively, reduces the number of cells surviving to the immature neuron stage in the DG, and that a paucity of PV+ interneurons could be contributing to this.

### Aberrant neurodevelopment

**Figure 6:**
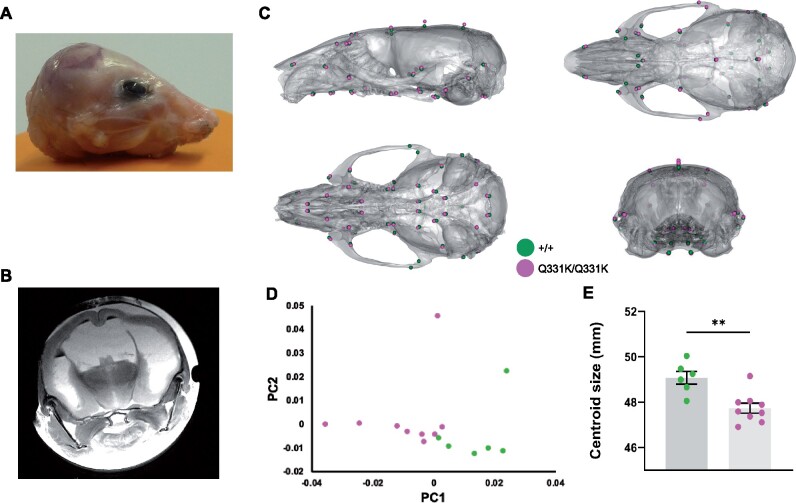
**Altered skull shape in TDP-43^Q331K/Q331K^ mice.** (A) Photo of scalped head of a Q331K/Q331K mouse and (**B**) *ex vivo* MRI scan of fixed head of the mouse. (**C**) Geometric morphometric analysis of +/+ versus Q331K/Q331K mice. Average landmark configurations of +/+ and Q331K/Q331K skulls superimposed on +/+ average skull, showing (clockwise from top left) lateral, superior, rear and inferior views. Each dot represents a single standardized landmark, the colour indicating the average for each genotype. (**D**) Principal component analysis (first two components) of skull shape variation after scaling. (**E**) Centroid sizes (the square root of the sum of the squared distances of a set of landmarks from their collective centroid) of +/+ and Q331K/Q331K skulls. Groups were compared by unpaired two-tailed *t*-test: ** *P* = 0.0022. Error bars represent mean ± SEM. (**D, E**) Each dot represents one mouse.

One of the most striking observations on MRI was the lateral ventricular enlargement in TDP-43^Q331K/Q331K^ mice. This could simply be secondary to a reduction in the brain parenchyma, but another explanation is that the ventricular system had not developed properly and that the ventriculomegaly actually represents a state of hydrocephalus. Indeed, the cerebral aqueduct was significantly reduced in volume in mutants (Table 1), indicating that cerebrospinal fluid outflow could be compromised. Furthermore, occasional juvenile mutants demonstrated frank hydrocephalus with doming of the skull and had to be euthanized before 6 weeks of age (Fig. 6A and B). We postulated that the ventricular enlargement of mutants that survived through to later adulthood could also have occurred early in life, before significant ossification of cranial sutures, and that this would be reflected as a change in skull shape that would persist into adulthood once skull plates had fused. We therefore performed *ex vivo* micro-CT on heads of aged 24-month-old male mice. Indeed, geometric morphometric comparison of TDP-43^Q331K/Q331K^ and wild-type littermate skulls using principal component analysis revealed a significant difference in shape between the two genotypes (Fig. 6C and D). Specifically, a subtly dysmorphic craniofacial structure was seen in mutants characterized by mid-facial hypoplasia and brachycephaly—effectively an increase in skull sphericity—and a reduction in overall skull size (Fig. 6E). These changes are in keeping with the hypothesis that TDP-43^Q331K^ disrupts the development of the ventricular system.

**Figure 7:**
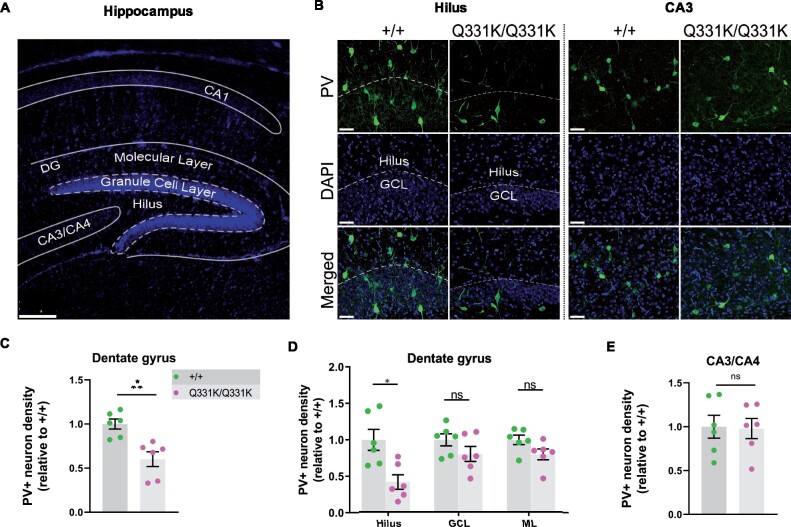
**Paucity of PV+ neurons in hippocampi of P14 TDP-43^Q331K/Q331K^ mice.** (A) Low magnification DAPI-stained overview of mouse P14 hippocampal regions. Scale bar, 200 μm. (**B**) PV+ interneurons shown in given regions of the hippocampus in P14 +/+ and Q331K/Q331K mice. Scale bar, 50 μm. (**C**) Quantification of PV+ interneuron density in dentate gyrus (DG) comparing +/+ to Q331K/Q331K mice: * *P* = 0.0142. (**D**) Of regions within DG, quantification of PV+ interneuron density in hilus comparing +/+ to Q331K/Q331K mice: * *P* = 0.0315; granule cell layer (GCL): ns *P* = 0.3133 and molecular layer (ML): ns *P* = 0.1957. (**E**) Quantification of PV+ interneuron density in C3 area: ns *P* = 0.9061. (**C, D**) Each dot represents one mouse. *P* values were calculated with multiple *t*-tests adjusted by Holm–Sidak correction. Error bars represent mean ± SEM.

We also wished to determine if mutant TDP-43^Q331K^ influenced the development of the brain parenchyma itself. We therefore turned to our histological readout of choice, PV+ interneuron density, looking specifically at the hippocampi of juvenile P14 mouse pups (Fig. 7A). We found a significant reduction of PV+ interneuron density in the DG of mutant mice, particularly the hilus, but no changes in the CA3 region (Fig. 7B–E). These observations establish a role for TDP-43 in the development of PV+ interneurons in the brain. This in turn suggests that the paucity of PV+ interneurons in adult mice is at least partly due to their failure to develop in the first place.

### Paucity of PV+ interneurons in human ALS

**Figure 8:**
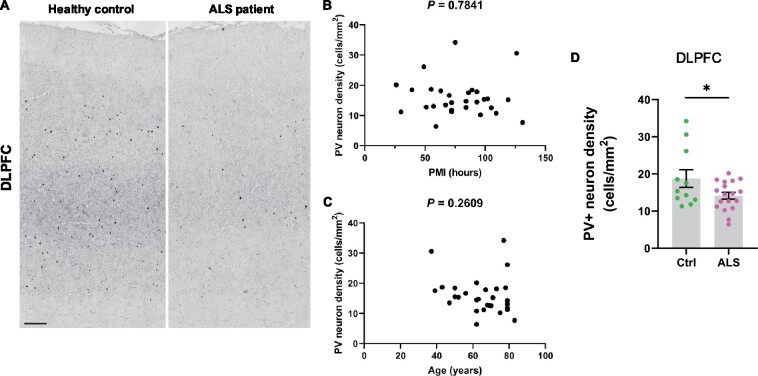
**Paucity of PV+ neurons in DLPFC of ALS patients.** (A) Immunohistochemistry showing PV+ interneurons in the dorsolateral prefrontal cortex (DLPFC) of neurologically healthy controls and ALS patients. Scale bar, 300 μm. (**B**) Scatter plot demonstrating PV+ interneuron density versus post mortem interval for each case (cases and controls combined). (**C**) Scatter plot demonstrating PV+ interneuron density versus age of each patient (cases and controls combined). **D** Quantification of PV+ interneuron density (cells/mm^2^) in DLPFC: * *P* = 0.0447. Each dot represents one case. Groups were compared by unpaired two-tailed *t*-test: Error bars represent mean ± SEM.

Our MRI-guided histological survey in mutant mice indicated a central role for PV+ interneurons in several aspects of TDP-43^Q331K^-mediated disease: regional vulnerability of the brain, aberrant neurogenesis and neurodevelopment. We therefore sought to determine if disruption to PV+ interneurons was a feature of human disease. We immunostained for PV in the dorsolateral prefrontal cortex of 18 patients with ALS patients (6 with *C9orf72* mutations and 12 sporadic ALS cases without known ALS gene mutations) and 11 age and sex-matched neurologically normal controls (Fig. 8A). Demographic details of brain donors are given in Supplementary Tables 2 and 3, including post-mortem interval, sex, age and *C9orf72* genetic status. There was no significant effect of post-mortem interval or age on PV+ interneuron density (Fig. 8B and C). However, we detected a 24.6% reduction of PV+ interneuron density in ALS patients relative to controls (Fig. 8A and D) with both mutant *C9orf72*-linked ALS and sporadic ALS cases showing a similar deficiency. This confirms that a paucity of PV+ interneurons in the frontal cortex is a feature of human ALS. Braak and Thal staging of the frontal cortex of a subset of cases did not suggest that tau or amyloid pathology was associated with PV neuron loss (Supplementary Tables 2 and 3), though more studies are needed to confirm this.

## Discussion

Treatment for neurodegenerative diseases is likely to be most effective during presymptomatic or prodromal stages, and biomarkers sensitive to these periods are needed. Preclinical models that accurately reflect human disease are therefore essential, facilitating the discovery of molecular pathological changes underlying the very earliest stages of disease. Here, using structural MRI, we detected an extensive pattern of brain volume loss in TDP-43^Q331K^ knock-in mice, a preclinical model for ALS-FTD, while still at an early stage of disease. Significantly, many of the brain areas affected in mutant mice are analogous to those involved in patients with FTD.^63^^,^^64^ Similar regions have also been implicated in recent studies of patients with ALS.^3^ Perhaps most importantly, presymptomatic carriers of ALS-FTD-linked gene mutations also demonstrate atrophy in these brain areas, even decades in advance of clinical onset.^2^^,^^4^^,^^37^ A detailed list of affected mouse and human brain regions is given in Table 1. Collectively, these observations underscore the value of the TDP-43^Q331K^ knock-in mouse in revealing *in vivo* structural biomarkers relevant to the pathogenesis of disease in patients with ALS-FTD.

Interestingly, our MRI studies of TDP-43^Q331K^ mice highlighted numerous regions beyond the frontal and temporal lobes that have only recently also emerged as being affected in patients. Thalamic regions were smaller in mutant mice, mirroring observations in patients with sporadic and familial FTD caused by mutations in *C9orf72*, *MAPT* and *GRN*. The thalamus represents an important hub, modulating the flow of information between the external environment and the cortex, and could be contributing to many facets of FTD including abnormalities of cognition, attention and personality. The cerebellum was also affected in mutant mice, and it has been shown that cerebellar atrophy occurs in both mutant *C9orf72* and *MAPT* associated FTD.^2^ Furthermore, cerebellar integrity has been associated with cognitive performance,^52^ while functional MRI has identified impaired connectivity of cerebellar regions with the cerebrum in FTD.^65^ Dysfunctional cerebellar and cortico-cerebellar circuits, particularly involving the vermis and the deep nuclei, are recognized as core contributors to psychosis, which can be part of the clinical picture of FTD.^66^ Finally, the flocculus, which functions in ocular motility, was also affected in mutant mice, and this is notable as MRI changes in the cerebellum and eye movement abnormalities can occur in patients with ALS.^67^^,^^68^ More detailed studies of cerebellar changes in TDP-43^Q331K^ mice may help to unravel the role of this intriguing brain structure in the symptomatology and pathogenesis of ALS-FTD.

Enlargement of the lateral ventricles is also emerging as a sensitive MRI biomarker in human FTD.^25^^,^^54^ This may be due to subcortical parenchymal volume loss since one of the imaging hallmarks of FTD, and a point of divergence from Alzheimer’s disease (AD), appears to be greater subcortical than cortical brain atrophy.^69^ It was therefore remarkable that TDP-43^Q331K^ mice also had enlarged lateral ventricles. While this ventriculomegaly could be due to loss of brain parenchyma, it could also reflect hydrocephalus. In this respect, it is notable that elderly patients with FTD and concomitant hydrocephalus can show clinical improvement following cerebrospinal fluid removal.^70^^,^^71^ Assaying cerebrospinal fluid pressure in TDP-43^Q331K^ mice would therefore be of interest. Nonetheless, our observation that frank hydrocephalus occurred in a small number of juvenile TDP-43^Q331K^ mice is enough to suggest that a developmental contribution towards ventricular enlargement in mutants is likely. Corroborating this, our micro-CT analysis elucidated that even aged TDP-43^Q331K^ mice have more spherical skulls, suggesting that intracranial pressure was raised prior to skull plate fusion (i.e. during development). Although skull shape changes can occur in humans in association with age-related brain atrophy,^72^ we conclude that enlargement of ventricles in TDP-43^Q331K^ mice is at least partly developmental in origin. A dedicated neuroimaging study of younger mice with serial scans as they age would be of value to determine to what extent structural changes are developmental or degenerative.

The recapitulation in TDP-43^Q331K^ knock-in mice of analogous brain MRI changes seen in prodromal human ALS-FTD allowed us to address a fundamental question in neurology: how do disturbances in ubiquitously expressed proteins such as TDP-43 cause regionally selective degeneration in the nervous system? We approached this question by performing a histological survey and found that TDP-43^Q331K^ knock-in mice were deficient in PV+ interneurons in areas showing atrophy on MRI, but not in an area with no volume loss. While this finding remains an association, it is notable that we also found that PV+ interneurons are reduced in human ALS frontal cortex. Previous studies of cortical GABAergic interneurons in ALS have identified robust reductions in calbindin D-28 immunoreactive cells in the motor cortex,^55^^,^^56^ but analyses of PV+ interneuron numbers have been less conclusive. One study found a significant decrease of PV+ interneurons in the motor cortex but did not examine more anterior regions of the brain,^57^ while another found only a trend towards a reduction in layer VI of the frontal cortex.^56^ A third study found no decrease in PV+ interneurons in the frontal cortex in two patients with ALS with dementia.^55^ Our study more strongly points towards a reduction in PV+ interneurons as being correlated with ALS and, furthermore, shows that both sALS and *C9orf72*-linked ALS display a similar degree of loss. In theory, such a loss of PV+ interneurons could lead to a reduction in cortical inhibition, which could have excitotoxic consequences for the pyramidal projection neurons onto which they synapse.^73^ In support of this proposal, patients with mutations in *C9orf72*, the most common genetic cause of ALS and FTD, have been found to demonstrate cortical hyperexcitability.^74^ Other mouse models of ALS have also identified a reduction in PV+ interneurons, including transgenic wild-type TDP-43 and SOD1^G93A^ mice.^75^^,^^76^ Future studies should aim to determine whether the reduced PV staining is due to a paucity of neurons or, alternatively, a reduction in PV expression within a subset of PV neurons (possibly due to a reduction in their activity). It will also be of value to determine if PV neuron loss is seen more broadly in other genetic forms of ALS and FTD. Further immunohistochemical studies of larger cohorts of patients are needed. Nonetheless, aiming to promote PV+ interneuron activity is worthy of further study as a possible disease-modifying approach in neurodegenerative disease. The relevance of this approach could be broad as PV neuron loss has been observed in Huntington’s disease, Lewy body disease and Alzheimer’s disease.^77–79^

Intriguingly, we also observed a paucity of PV+ interneurons in P14 mice. This suggests that the deficiency of these cells in adult mice may be because they did not develop in the first place. Fast-spiking PV+ interneurons are the main modulators of synaptic plasticity during the critical period of cortical development, and disruption of interneurons leads to abnormalities in neuronal excitability, a feature of neurodevelopmental disorders, such as autism and schizophrenia.^80–83^ While ALS-FTD is not regarded as a developmental disease, mutations in some ALS-causing genes have been associated with juvenile-onset ALS with learning difficulties and autism,^84^^,^^85^ and studies have suggested that network degeneration in asymptomatic carriers of *C9orf72* mutations could be a consequence of aberrant patterning during development.^38^ There is also growing evidence of a genetic correlation between ALS and schizophrenia, another disease of neurodevelopmental origin.^86^^,^^87^ In this respect, it is interesting to note that both autism and schizophrenia demonstrate a paucity of PV+ interneurons.^83^ Thus, it is not inconceivable that disturbances in cortical excitation–inhibition balance during development may later predispose an individual to developing an age-related neurodegenerative disease. More studies are needed to map the migration and proliferation of embryonic PV neurons to determine to what extent their paucity in the adult mouse brain is developmental or degenerative. Human derived brain organoids could be a useful approach to validate in man.

Another important role served by PV+ interneurons is that of promoting hippocampal neurogenesis in the adult brain.^62^ We were therefore intrigued by the observation that the DG of the hippocampus, a niche for AHN, was smaller in TDP-43^Q331K^ mice and demonstrated a significant loss of PV+ interneurons. Examination of the number of immature neurons suggested that AHN was impaired in mutant mice. An alternative intriguing notion is the possibility that the reduction in DCX neurons is due to accelerated maturation as a compensatory response to neuronal loss. Validation with birth-dating techniques (e.g. using thymidine analogues such as BrdU or EdU) used in parallel with markers of neurons (NeuN) would be helpful to confirm a role for TDP-43 in neurogenesis.

While AHN has been shown to be impaired in AD,^88^^,^^89^ neurogenesis has been little studied in ALS-FTD. Interestingly, however, one human post-mortem study identified a reduction in the proliferation of neural progenitor cells in the hippocampi of patients with ALS.^90^ Furthermore, overexpression of TDP-43 in neural progenitor cells of the developing mouse telencephalon was found to impair neurogenesis,^91^ while knockdown of TDP-43 *in vitro* resulted in increased expression of neurogenin 2, a transcription factor that specifies neuronal fate during development.^92^ Our study adds to this literature by demonstrating an *in vivo* role for TDP-43 in neurogenesis within the adult brain. Given the importance of adult-born hippocampal neurons to spatial memory, pattern separation, mood and cognition,^93^^,^^94^ impaired AHN due to TDP-43 misregulation may well have contributed to the behavioural phenotypes we previously described in mutant mice.^13^ Further studies to delineate the link between PV+ interneurons and AHN are warranted as we speculate that this will help find ways to leverage the neurogenic potential of the brain for therapeutic benefit in ALS-FTD.

In our analysis of non-neuronal cells we noted that microglia were in an activated state in TDP-43^Q331K^ knock-in mice. Microglia are thought to have a ‘surveying’ role when quiescent and become activated in neurodegenerative diseases, engulfing cellular debris, phagocytosing protein aggregates and pruning synapses, possibly through complement activation.^95–97^ Using inducible TDP-43 transgenic mice, it has been shown that microglia play a key role in recovery from neurodegeneration.^98^ Unexpectedly, we found that microglial activation occurred even in the visual cortex, an area that was largely unaffected on MRI and which was not deficient in PV+ interneurons. This suggests that microglial activation is widespread and does not explain regional brain vulnerability in TDP-43^Q331K^ knock-in mice, at least when 7 months old. This also raises the intriguing possibility that microglial activation precedes and therefore may cause neuronal loss. Although activated microglia in the mouse brain is regionally and functionally heterogeneous, and they may not be acting destructively, the reduced Tmem119 we observed is indicative of a shift to a DAM phenotype in mutants. This prompted us to re-examine our previous RNASeq analysis of frontal cortical tissue from 5- to 20-month-old mice^13^ (Supplementary Table 4). Looking specifically for microglial and microglia-related genes that were differentially expressed between wild-type and homozygous mutant mice, we confirmed that *Iba1* gene expression was increased. Intriguingly, *Tmem119* gene expression was downregulated in mutants, which contrasts with the increase in Tmem119 protein that we found with immunohistochemistry. This dissociation between *Tmem119* transcript and protein levels has been noted before in the context of activated microglia.^18^ Further, work should therefore be directed at examining how protein translation is altered in activated microglia as well as more detailed analyses of microglial morphology in TDP-43^Q331K^ mutant mice.

## Conclusions

In conclusion, MRI changes in human ALS-FTD are recapitulated in TDP-43^Q331K^ knock-in mice, allowing this model to be used to understand the cellular and molecular meaning of early macroscopic brain changes, something that is currently impossible in humans. Further studies of TDP-43^Q331K^ knock-in mice (including female mice) using longitudinal structural and functional MRI and MR spectroscopy promise to help delineate the natural history of ALS-FTD, and the role of neurodevelopmental abnormalities in disease. More detailed examination of molecular changes within affected and unaffected brain regions, for example using *in situ* transcriptomic approaches, should help to unravel how different neuronal and glial subtypes contribute to disease, including during development and in neurogenic niches. This approach, in quantifying *TARDBP* transcript levels, may also help to determine the extent to which TDP-43 expression level correlates with volume loss. These profiles will ultimately be of value in measuring the efficacy of therapeutic approaches designed to treat and perhaps even prevent ALS-FTD.

## Supplementary material

Supplementary material is available at *Brain Communications* online.

## Competing interests

The authors report no competing interests.

## Funding

J. Sreedharan gratefully acknowledges support from the Motor Neuron Disease Association, the Medical Research Council UK (MR/K010611/1), the Lady Edith Wolfson Fellowship Fund, the van Geest Foundation, the Rosetrees Trust (M799), Alzheimer’s Research UK (ARUK-PG2018B-008), and the Psychiatry Research Trust. M.P.C is supported by the van Geest Foundation. We gratefully acknowledge the Chinese Scholarship Council for supporting Ziqiang Lin during this study. This work was supported by the Alzheimer’s Research UK King’s College London Network Centre.

## Supplementary Material

fcab114_Supplementary_Data

## Abbreviations

AD =: Alzheimer's disease
AHN =: adult hippocampal neurogenesis
ALS =: amyotrophic lateral sclerosis
DAB =: 3,3′-diaminobenzidine
DAM =: disease-associated microglia
DCX =: doublecortin
FTD =: frontotemporal dementia
FWE =: family-wise error
Iba-1 =: ionized calcium-binding adapter molecule 1
MND =: motor neurone disease
PV =: parvalbumin
ROI =: region of interest
RT =: room temperature
SOM =: somatostatin
TBM =: tensor-based morphometry
Tmem119 =: transmembrane protein 119

## References

[1] Ahmed RM , Irish M, Henning E, et al Assessment of eating behavior disturbance and associated neural networks in frontotemporal dementia. JAMA Neurol. 2016;73:282–290. 26810632 10.1001/jamaneurol.2015.4478

[2] Cash DM , Bocchetta M, Thomas DL, et al; Genetic FTD Initiative, GENFI. Patterns of gray matter atrophy in genetic frontotemporal dementia: Results from the GENFI study. Neurobiol Aging. 2018;62:191–196. 29172163 10.1016/j.neurobiolaging.2017.10.008 PMC5759893

[3] Menke RA , Agosta F, Grosskreutz J, Filippi M, Turner MR. Neuroimaging endpoints in amyotrophic lateral sclerosis. Neurotherapeutics. 2017;14:11–23. 27752938 10.1007/s13311-016-0484-9 PMC5233627

[4] Panman JL , Jiskoot LC, Bouts MJRJ, et al Gray and white matter changes in presymptomatic genetic frontotemporal dementia: A longitudinal MRI study. Neurobiol Aging. 2019;76:115–124. 30711674 10.1016/j.neurobiolaging.2018.12.017

[5] Bertrand A , Wen J, Rinaldi D, et al; Predict to Prevent Frontotemporal Lobar Degeneration and Amyotrophic Lateral Sclerosis (PREV-DEMALS) Study Group. Early cognitive, structural, and microstructural changes in presymptomatic C9orf72 carriers younger than 40 years. JAMA Neurol. 2018;75:236–245. 29197216 10.1001/jamaneurol.2017.4266 PMC5838615

[6] Thompson PM , Martin NG, Wright MJ. Imaging genomics. Curr Opin Neurol. 2010;23:368–373. 20581684 10.1097/WCO.0b013e32833b764c PMC2927195

[7] Burrell JR , Halliday GM, Kril JJ, et al The frontotemporal dementia-motor neuron disease continuum. Lancet. 2016;388:919–931. 26987909 10.1016/S0140-6736(16)00737-6

[8] Neumann M , Sampathu DM, Kwong LK, et al Ubiquitinated TDP-43 in frontotemporal lobar degeneration and amyotrophic lateral sclerosis. Science. 2006;314:130–133. 17023659 10.1126/science.1134108

[9] Arai T , Hasegawa M, Akiyama H, et al TDP-43 is a component of ubiquitin-positive tau-negative inclusions in frontotemporal lobar degeneration and amyotrophic lateral sclerosis. Biochem Biophys Res Commun. 2006;351:602–611. 17084815 10.1016/j.bbrc.2006.10.093

[10] Sreedharan J , Blair IP, Tripathi VB, et al TDP-43 mutations in familial and sporadic amyotrophic lateral sclerosis. Science. 2008;319:1668–1672. 18309045 10.1126/science.1154584 PMC7116650

[11] Benajiba L , Le Ber I, Camuzat A, et al French Clinical and Genetic Research Network on Frontotemporal Lobar Degeneration/Frontotemporal Lobar Degeneration with Motoneuron Disease. TARDBP mutations in motoneuron disease with frontotemporal lobar degeneration. Ann Neurol. 2009;65:470–473. 19350673 10.1002/ana.21612

[12] Wu LS , Cheng WC, Hou SC, Yan YT, Jiang ST, Shen CK. TDP-43, a neuro-pathosignature factor, is essential for early mouse embryogenesis. Genesis. 2010;48:56–62. 20014337 10.1002/dvg.20584

[13] White MA , Kim E, Duffy A, et al TDP-43 gains function due to perturbed autoregulation in a Tardbp knock-in mouse model of ALS-FTD. Nat Neurosci. 2018;21:552–563. 29556029 10.1038/s41593-018-0113-5 PMC5884423

[14] Dorr AE , Lerch JP, Spring S, Kabani N, Henkelman RM. High resolution three-dimensional brain atlas using an average magnetic resonance image of 40 adult C57Bl/6J mice. Neuroimage. 2008;42:60–69. 18502665 10.1016/j.neuroimage.2008.03.037

[15] Satoh J , Kino Y, Asahina N, et al TMEM119 marks a subset of microglia in the human brain. Neuropathology. 2016;36:39–49. 26250788 10.1111/neup.12235

[16] Butovsky O , Jedrychowski MP, Moore CS, et al Identification of a unique TGF-beta-dependent molecular and functional signature in microglia. Nat Neurosci. 2014;17:131–143. 24316888 10.1038/nn.3599 PMC4066672

[17] Deczkowska A , Keren-Shaul H, Weiner A, Colonna M, Schwartz M, Amit I. Disease-associated microglia: A universal immune sensor of neurodegeneration. Cell. 2018;173:1073–1081. 29775591 10.1016/j.cell.2018.05.003

[18] Bennett ML , Bennett FC, Liddelow SA, et al New tools for studying microglia in the mouse and human CNS. Proc Natl Acad Sci USA. 2016;113:E1738–46. 26884166 10.1073/pnas.1525528113 PMC4812770

[19] White MA , Lin Z, Kim E, et al Sarm1 deletion suppresses TDP-43-linked motor neuron degeneration and cortical spine loss. Acta Neuropathol Commun. 2019;7:166. 31661035 10.1186/s40478-019-0800-9 PMC6819591

[20] Hallgrimsson B , Lieberman DE, Liu W, Ford-Hutchinson AF, Jirik FR. Epigenetic interactions and the structure of phenotypic variation in the cranium. Evol Dev. 2007;9:76–91. 17227368 10.1111/j.1525-142X.2006.00139.x

[21] Toussaint N , Redhead Y, Liu W, et al Application of high-resolution landmark-free morphometrics to a mouse model of Down Syndrome reveals a tightly localised cranial phenotype. bioRxiv. 2019;19:1053–1035.

[22] Brooks BR , Miller RG, Swash M, Munsat TL; World Federation of Neurology Research Group on Motor Neuron Diseases. El Escorial revisited: Revised criteria for the diagnosis of amyotrophic lateral sclerosis. Amyotroph Lateral Scler Other Motor Neuron Disord. 2000;1:293–299. 11464847 10.1080/146608200300079536

[23] The Mouse Imaging Centre THfSC, Toronto. https://wiki.mouseimaging.ca/display/MICePub/Mouse+Brain+Atlases#MouseBrainAtlases-Dorr-Steadman-Ullmann-Richards-Qiu-Egan(40micron,DSURQE), last accessed 7 June 2021.

[24] Thomann PA , Dos Santos V, Toro P, Schönknecht P, Essig M, Schröder J. Reduced olfactory bulb and tract volume in early Alzheimer's disease—A MRI study. Neurobiol Aging. 2009;30(5):838–841. 17875348 10.1016/j.neurobiolaging.2007.08.001

[25] Manera AL , Dadar M, Collins DL, Ducharme S; Frontotemporal Lobar Degeneration Neuroimaging Initiative. Deformation based morphometry study of longitudinal MRI changes in behavioral variant frontotemporal dementia. Neuroimage Clin. 2019;24:102079. 31795051 10.1016/j.nicl.2019.102079 PMC6879994

[26] Seeley WW , Crawford R, Rascovsky K, et al Frontal paralimbic network atrophy in very mild behavioral variant frontotemporal dementia. Arch Neurol. 2008;65:249–255. 18268196 10.1001/archneurol.2007.38 PMC2544627

[27] Boccardi M , Sabattoli F, Laakso MP, et al Frontotemporal dementia as a neural system disease. Neurobiol Aging. 2005;26:37–44. 15585344 10.1016/j.neurobiolaging.2004.02.019

[28] Devenney EM , Landin-Romero R, Irish M, et al The neural correlates and clinical characteristics of psychosis in the frontotemporal dementia continuum and the C9orf72 expansion. Neuroimage Clin 2017;13:439–445. 28116236 10.1016/j.nicl.2016.11.028 PMC5233794

[29] Borroni B , Alberici A, Cercignani M, et al Granulin mutation drives brain damage and reorganization from preclinical to symptomatic FTLD. Neurobiol Aging. 2012;33:2506–2520. 22130207 10.1016/j.neurobiolaging.2011.10.031

[30] Whitwell JL , Weigand SD, Boeve BF, et al Neuroimaging signatures of frontotemporal dementia genetics: C9ORF72, tau, progranulin and sporadics. Brain. 2012;135:794–806. 22366795 10.1093/brain/aws001 PMC3286334

[31] Muñoz-Ruiz MÁ , Hartikainen P, Koikkalainen J, et al Structural MRI in frontotemporal dementia: Comparisons between hippocampal volumetry, tensor-based morphometry and voxel-based morphometry. PLoS One. 2012;7:e52531-12- 23285078 10.1371/journal.pone.0052531 PMC3527560

[32] Bertoux M , O'Callaghan C, Flanagan E, Hodges JR, Hornberger M. Fronto-striatal atrophy in behavioral variant frontotemporal dementia and Alzheimer's disease. Front Neurol. 2015;6:147. 26191038 10.3389/fneur.2015.00147 PMC4486833

[33] Cardenas VA , Boxer AL, Chao LL, et al Deformation-based morphometry reveals brain atrophy in frontotemporal dementia. Arch Neurol. 2007;64:873–879. 17562936 10.1001/archneur.64.6.873 PMC2733361

[34] Menke RA , Proudfoot M, Wuu J, et al Increased functional connectivity common to symptomatic amyotrophic lateral sclerosis and those at genetic risk. J Neurol Neurosurg Psychiatry. 2016;87:580–588. 26733601 10.1136/jnnp-2015-311945 PMC4893149

[35] Westeneng H-J , Walhout R, Straathof M, et al Widespread structural brain involvement in ALS is not limited to the C9orf72repeat expansion. J Neurol Neurosurg Psychiatry. 2016;87:1354–1360. 27756805 10.1136/jnnp-2016-313959 PMC5136726

[36] Menke RAL , Proudfoot M, Talbot K, Turner MR. The two-year progression of structural and functional cerebral MRI in amyotrophic lateral sclerosis. Neuroimage Clin. 2018;17:953–961. 29321969 10.1016/j.nicl.2017.12.025 PMC5752097

[37] Rohrer JD , Nicholas JM, Cash DMMD ED, MSc LJ, et al Presymptomatic cognitive and neuroanatomical changes in genetic frontotemporal dementia in the Genetic Frontotemporal dementia Initiative (GENFI) study: A cross-sectional analysis. Lancet Neurol. 2015;14:253–262. 25662776 10.1016/S1474-4422(14)70324-2 PMC6742501

[38] Lee SE , Sias AC, Mandelli ML, et al Network degeneration and dysfunction in presymptomatic C9ORF72 expansion carriers. Neuroimage Clin. 2017;14:286–297. 28337409 10.1016/j.nicl.2016.12.006 PMC5349617

[39] Jiskoot LC , Panman JL, Meeter LH, et al Longitudinal multimodal MRI as prognostic and diagnostic biomarker in presymptomatic familial frontotemporal dementia. Brain. 2019;142:193–208. 30508042 10.1093/brain/awy288 PMC6308313

[40] Bede P , Bokde ALW, Byrne S, et al Multiparametric MRI study of ALS stratified for the C9orf72 genotype. Neurology. 2013;81:361–369. 23771489 10.1212/WNL.0b013e31829c5eee PMC3772833

[41] Olm CA , McMillan CT, Irwin DJ, et al Longitudinal structural gray matter and white matter MRI changes in presymptomatic progranulin mutation carriers. Neuroimage Clin. 2018;19:497–506. 29984158 10.1016/j.nicl.2018.05.017 PMC6029561

[42] Papma JM , Jiskoot LC, Panman JL, et al Cognition and gray and white matter characteristics of presymptomatic C9orf72 repeat expansion. Neurology. 2017;89:1256–1264. 28855404 10.1212/WNL.0000000000004393

[43] Bejanin A , Murray ME, Martin P, et al Antemortem volume loss mirrors TDP-43 staging in older adults with non-frontotemporal lobar degeneration. Brain. 2019;142:3621–3615. 31562527 10.1093/brain/awz277 PMC6821218

[44] Laakso MP , Frisoni GB, Könönen M, et al Hippocampus and entorhinal cortex in frontotemporal dementia and Alzheimer’s disease: A morphometric MRI study. Biol Psychiatry. 2000;47:1056–1063. 10862805 10.1016/s0006-3223(99)00306-6

[45] Eslinger PJ , Moore P, Antani S, Anderson C, Grossman M. Apathy in frontotemporal dementia: Behavioral and neuroimaging correlates. Behav Neurol. 2012;25:127–136. 22425723 10.3233/BEN-2011-0351 PMC3640327

[46] Westeneng HJ , Verstraete E, Walhout R, et al Subcortical structures in amyotrophic lateral sclerosis. Neurobiol Aging. 2015;36:1075–1082. 25281019 10.1016/j.neurobiolaging.2014.09.002

[47] Walhout R , Schmidt R, Westeneng HJ, et al Brain morphologic changes in asymptomatic C9orf72 repeat expansion carriers. Neurology. 2015;85:1780–1788. 26497991 10.1212/WNL.0000000000002135

[48] Halabi C , Halabi A, Dean DL, et al Patterns of striatal degeneration in frontotemporal dementia. Alzheimer Dis Assoc Disord. 2013;27:74–83. 22367382 10.1097/WAD.0b013e31824a7df4 PMC3389579

[49] Bocchetta M , Gordon E, Cardoso MJ, et al Thalamic atrophy in frontotemporal dementia — Not just a C9orf72 problem. Neuroimage Clin. 2018;18:675–681. 29876259 10.1016/j.nicl.2018.02.019 PMC5988457

[50] Floeter MK , Bageac D, Danielian LE, Braun LE, Traynor BJ, Kwan JY. Longitudinal imaging in C9orf72 mutation carriers: Relationship to phenotype. NeuroImage Clin. 2016;12:1035–1043. 27995069 10.1016/j.nicl.2016.10.014 PMC5153604

[51] Kilimann I , Hausner L, Fellgiebel A, et al Parallel atrophy of cortex and basal forebrain cholinergic system in mild cognitive impairment. Cerebral Cortex. 2016;23:bhw019–8. 10.1093/cercor/bhw019 26879092

[52] Chen Y , Kumfor F, Landin-Romero R, Irish M, Hodges JR, Piguet O. Cerebellar atrophy and its contribution to cognition in frontotemporal dementias. Ann Neurol. 2018;84:98–109. 30014499 10.1002/ana.25271

[53] Gellersen HM , Guo CC, Apos O, et al Cerebellar atrophy in neurodegeneration-a meta-analysis. J Neurol Neurosurg Psychiatry. 2017;88:780–788. 28501823 10.1136/jnnp-2017-315607

[54] Tavares TP , Mitchell DGV, Coleman K, et al; Genetic FTD Initiative, GENFI. Ventricular volume expansion in presymptomatic genetic frontotemporal dementia. Neurology. 2019;93:e1699–e706. 31578297 10.1212/WNL.0000000000008386 PMC6946476

[55] Ferrer I , Tunon T, Serrano MT, et al Calbindin D-28k and parvalbumin immunoreactivity in the frontal cortex in patients with frontal lobe dementia of non-Alzheimer type associated with amyotrophic lateral sclerosis. J Neurol Neurosurg Psychiatry. 1993;56:257–261. 8459241 10.1136/jnnp.56.3.257 PMC1014857

[56] Maekawa S , Al-Sarraj S, Kibble M, et al Cortical selective vulnerability in motor neuron disease: A morphometric study. Brain. 2004;127:1237–1251. 15130949 10.1093/brain/awh132

[57] Nihei K , McKee AC, Kowall NW. Patterns of neuronal degeneration in the motor cortex of amyotrophic lateral sclerosis patients. Acta Neuropathol. 1993;86:55–61. 8396837 10.1007/BF00454899

[58] Radford RA , Morsch M, Rayner SL, Cole NJ, Pountney DL, Chung RS. The established and emerging roles of astrocytes and microglia in amyotrophic lateral sclerosis and frontotemporal dementia. Front Cell Neurosci. 2015;9:414- 26578880 10.3389/fncel.2015.00414 PMC4621294

[59] Umoh ME , Dammer EB, Dai J, et al A proteomic network approach across the ALS-FTD disease spectrum resolves clinical phenotypes and genetic vulnerability in human brain. EMBO Mol Med. 2018;10:48–62. 29191947 10.15252/emmm.201708202 PMC5760858

[60] Neumann M , Kwong LK, Truax AC, et al TDP-43-positive white matter pathology in frontotemporal lobar degeneration with ubiquitin-positive inclusions. J Neuropathol Exp Neurol. 2007;66:177–183. 17356379 10.1097/01.jnen.0000248554.45456.58

[61] Groisman AI , Yang SM, Schinder AF. Differential coupling of adult-born granule cells to parvalbumin and somatostatin interneurons. Cell Rep. 2020;30:202–214 e4. 31914387 10.1016/j.celrep.2019.12.005 PMC7011182

[62] Song J , Sun J, Moss J, et al Parvalbumin interneurons mediate neuronal circuitry – neurogenesis coupling in the adult hippocampus. Nat Neurosci. 2013;16:1728–1730. 24212671 10.1038/nn.3572 PMC4096812

[63] Meeter LH , Kaat LD, Rohrer JD, van Swieten JC. Imaging and fluid biomarkers in frontotemporal dementia. Nat Rev Neurol. 2017;13:406–419. 28621768 10.1038/nrneurol.2017.75

[64] Bruun M , Koikkalainen J, Rhodius-Meester HFM, et al Detecting frontotemporal dementia syndromes using MRI biomarkers. NeuroImage Clin. 2019;22:101711–101719. 30743135 10.1016/j.nicl.2019.101711 PMC6369219

[65] Guo CC , Tan R, Hodges JR, Hu X, Sami S, Hornberger M. Network-selective vulnerability of the human cerebellum to Alzheimer’s disease and frontotemporal dementia. Brain. 2016;139:1527–1538. 26912642 10.1093/brain/aww003 PMC5839595

[66] Schmahmann JD. Cerebellum in Alzheimer's disease and frontotemporal dementia: Not a silent bystander. Brain. 2016;139:1314–1318. 27189578 10.1093/brain/aww064

[67] Prell T , Grosskreutz J. The involvement of the cerebellum in amyotrophic lateral sclerosis. Amyotroph Lateral Scl Frontotemporal Degener. 2013;14:507–515. 10.3109/21678421.2013.812661 23889583

[68] Kang BH , Kim JI, Lim YM, Kim KK. Abnormal oculomotor functions in amyotrophic lateral sclerosis. J Clin Neurol. 2018;14:464–471. 30198218 10.3988/jcn.2018.14.4.464 PMC6172508

[69] Landin-Romero R , Kumfor F, Leyton CE, Irish M, Hodges JR, Piguet O. Disease-specific patterns of cortical and subcortical degeneration in a longitudinal study of Alzheimer's disease and behavioural-variant frontotemporal dementia. Neuroimage. 2017;151:72–80. 27012504 10.1016/j.neuroimage.2016.03.032

[70] Korhonen VE , Solje E, Suhonen NM, et al Frontotemporal dementia as a comorbidity to idiopathic normal pressure hydrocephalus (iNPH): A short review of literature and an unusual case. Fluids Barriers CNS. 2017;14:10. 28420385 10.1186/s12987-017-0060-7 PMC5395836

[71] Korhonen VE , Remes AM, Helisalmi S, et al Prevalence of C9ORF72 expansion in a large series of patients with idiopathic normal-pressure hydrocephalus. Dement Geriatr Cogn Disord. 2019;47:91–103. 30861516 10.1159/000497306

[72] Urban JE , Weaver AA, Lillie EM, Maldjian JA, Whitlow CT, Stitzel JD. Evaluation of morphological changes in the adult skull with age and sex. J Anat. 2016;229:838–846. 25406956 10.1111/joa.12247 PMC5108156

[73] Kiernan MC , Ziemann U, Eisen A. Amyotrophic lateral sclerosis: Origins traced to impaired balance between neural excitation and inhibition in the neonatal period. Muscle Nerve. 2019;60:232–235. 31233613 10.1002/mus.26617

[74] Geevasinga N , Menon P, Yiannikas C, Kiernan MC, Vucic S. Diagnostic utility of cortical excitability studies in amyotrophic lateral sclerosis. Eur J Neurol. 2014;21:1451–1457. 24698287 10.1111/ene.12422

[75] Tsuiji H , Inoue I, Takeuchi M, et al TDP-43 accelerates age-dependent degeneration of interneurons. Sci Rep. 2017;7:14972. 29097807 10.1038/s41598-017-14966-w PMC5668320

[76] Quarta E , Fulgenzi G, Bravi R, et al Deletion of the endogenous TrkB.T1 receptor isoform restores the number of hippocampal CA1 parvalbumin-positive neurons and rescues long-term potentiation in pre-symptomatic mSOD1(G93A) ALS mice. Mol Cell Neurosci. 2018;89:33–41. 29580900 10.1016/j.mcn.2018.03.010 PMC8108068

[77] Bernstein HG , Johnson M, Perry RH, et al Partial loss of parvalbumin-containing hippocampal interneurons in dementia with Lewy bodies. Neuropathology. 2011;31:1–10. 20487308 10.1111/j.1440-1789.2010.01117.x

[78] Mehler MF , Petronglo JR, Arteaga-Bracho EE, et al Loss-of-Huntingtin in medial and lateral ganglionic lineages differentially disrupts regional interneuron and projection neuron subtypes and promotes Huntington's disease-associated behavioral, cellular, and pathological hallmarks. J Neurosci. 2019;39:1892–1909. 30626701 10.1523/JNEUROSCI.2443-18.2018 PMC6407290

[79] Richetin K , Steullet P, Pachoud M, et al Tau accumulation in astrocytes of the dentate gyrus induces neuronal dysfunction and memory deficits in Alzheimer's disease. Nat Neurosci. 2020;23:1567–1579. 33169029 10.1038/s41593-020-00728-x

[80] Tremblay R , Lee S, Rudy B. GABAergic interneurons in the neocortex: From cellular properties to circuits. Neuron. 2016;91:260–292. 27477017 10.1016/j.neuron.2016.06.033 PMC4980915

[81] Hu H , Gan J, Jonas P. Interneurons. Fast-spiking, parvalbumin(+) GABAergic interneurons: From cellular design to microcircuit function. Science. 2014;345:1255263- 25082707 10.1126/science.1255263

[82] Marin O. Developmental timing and critical windows for the treatment of psychiatric disorders. Nat Med. 2016;22:1229–1238. 27783067 10.1038/nm.4225

[83] Marín O. Interneuron dysfunction in psychiatric disorders. Nat Rev Neurosci. 2012;13:107–120. 22251963 10.1038/nrn3155

[84] Picher-Martel V , Brunet F, Dupre N, Chrestian N. The occurrence of FUS mutations in pediatric amyotrophic lateral sclerosis: A case report and review of the literature. J Child Neurol. 2020;35:556–562. 32281455 10.1177/0883073820915099

[85] Eura N , Sugie K, Suzuki N, et al A juvenile sporadic amyotrophic lateral sclerosis case with P525L mutation in the FUS gene: A rare co-occurrence of autism spectrum disorder and tremor. J Neurol Sci. 2019;398:67–68. 30684766 10.1016/j.jns.2019.01.032

[86] Vázquez-Costa JF , Beltrán E, Sopena P, et al Clinical and neuroimaging characterization of two C9orf72-positive siblings with amyotrophic lateral sclerosis and schizophrenia. Amyotroph Lateral Scl Frontotemporal Degener. 2016;17:297–300. 10.3109/21678421.2015.1112407 26613114

[87] McLaughlin RL , Schijven D, van Rheenen W, et al; Schizophrenia Working Group of the Psychiatric Genomics Consortium. Genetic correlation between amyotrophic lateral sclerosis and schizophrenia. Nat Commun. 2017;8:14774- 28322246 10.1038/ncomms14774 PMC5364411

[88] Moreno-Jimenez EP , Flor-Garcia M, Terreros-Roncal J, et al Adult hippocampal neurogenesis is abundant in neurologically healthy subjects and drops sharply in patients with Alzheimer's disease. Nat Med. 2019;25:554–560. 30911133 10.1038/s41591-019-0375-9

[89] Choi SH , Bylykbashi E, Chatila ZK, et al Combined adult neurogenesis and BDNF mimic exercise effects on cognition in an Alzheimer's mouse model. Science. 2018;361:eaan8821. 30190379 10.1126/science.aan8821 PMC6149542

[90] Galan L , Gomez-Pinedo U, Guerrero A, Garcia-Verdugo JM, Matias-Guiu J. Amyotrophic lateral sclerosis modifies progenitor neural proliferation in adult classic neurogenic brain niches. BMC Neurol. 2017;17:173. 28874134 10.1186/s12883-017-0956-5 PMC5585932

[91] Vogt MA , Ehsaei Z, Knuckles P, et al TDP-43 induces p53-mediated cell death of cortical progenitors and immature neurons. Sci Rep. 2018;8:8097. 29802307 10.1038/s41598-018-26397-2 PMC5970242

[92] Di Carlo V , Grossi E, Laneve P, et al TDP-43 regulates the microprocessor complex activity during in vitro neuronal differentiation. Mol Neurobiol. 2013;48:952–963. 24113842 10.1007/s12035-013-8564-x

[93] Luna VM , Anacker C, Burghardt NS, et al Adult-born hippocampal neurons bidirectionally modulate entorhinal inputs into the dentate gyrus. Science. 2019;364:578–583. 31073064 10.1126/science.aat8789 PMC6800071

[94] Sahay A , Scobie KN, Hill AS, O'Carroll CM, et al Increasing adult hippocampal neurogenesis is sufficient to improve pattern separation. Nature. 2011;472:466–470. 21460835 10.1038/nature09817 PMC3084370

[95] Wake H , Moorhouse AJ, Miyamoto A, Nabekura J. Microglia: Actively surveying and shaping neuronal circuit structure and function. Trends Neurosci. 2013;36:209–217. 23260014 10.1016/j.tins.2012.11.007

[96] Hong S , Beja-Glasser VF, Nfonoyim BM, et al Complement and microglia mediate early synapse loss in Alzheimer mouse models. Science. 2016;352:712–716. 27033548 10.1126/science.aad8373 PMC5094372

[97] Paolicelli RC , Jawaid A, Henstridge CM, et al TDP-43 depletion in microglia promotes amyloid clearance but also induces synapse loss. Neuron. 2017;95:297–308 e6. 28669544 10.1016/j.neuron.2017.05.037 PMC5519492

[98] Spiller KJ , Restrepo CR, Khan T, et al Microglia-mediated recovery from ALS-relevant motor neuron degeneration in a mouse model of TDP-43 proteinopathy. Nat Neurosci. 2018;21:329–340. 29463850 10.1038/s41593-018-0083-7 PMC5857237

