# Supplementary material for: MRI-guided histology of TDP-43 knock-in mice implicates parvalbumin interneuron loss, impaired neurogenesis and aberrant neurodevelopment in amyotrophic lateral sclerosis-frontotemporal dementia
Source: Brain Commun. 2021 May 27;3(2):fcab114. doi: 10.1093/braincomms/fcab114 (PMC8204366; doi:10.1093/braincomms/fcab114)
Supplement: fcab114_Supplementary_Data [file fcab114_supplementary_data.pdf]

Supplementary Table 1. Antibodies used in immunohistochemistry.

Protein	Primary Ab (product No.)	Primary Ab (dilution)	Secondary Ab (product No.)	Secondary Ab (dilution)
PV	Mouse, Sigma P3088	1:200	Goat, Alexa Fluor 488, Thermo Fisher	1:500
	Rabbit, Abcam ab11427	1:1000	Goat, Alexa Fluor 488, Thermo Fisher	1:500
GAD67	Mouse, Chemicon MAB5406	1:1000	Donkey, Alexa 568, Thermo Fisher	1:500
Iba1	Goat, Abcam ab5076	1:500	Donkey, Alexa Fluor 488, Thermo Fisher	1:500
Tmem119	Rabbit, Abcam ab209064	1:1000	Goat, Alexa Fluor 568, Thermo Fisher	1:500
Ki67	Rabbit, Abcam ab16667	1:500	Goat, biotinylated, Vector Lab BA1000	1:400
DCX	Goat, Santa Cruz Biotechnology	1:200	Horse, biotinylated, Vector Lab BA9500	1:400
SOM	Rat, Chemicon YC7	1:100	Goat, biotinylated, Vector Lab BA9401	1:500
MBP	Rat, Abcam ab7349	1:1000	Goat, biotinylated, Vector Lab BA9401	1:500
GFAP	Rabbit, Dako Z0334	1:2000	Goat, biotinylated, Vector Lab BA1000	1:1000

Supplementary Table 2 MRC numbers and conditions of patients.

ALS patients							
**MRC numbers**	**PMI (hrs)**	**Age (yrs)**	**Sex**	**Genetics**	**Braak stage**	**Thal stage**	**pTDP-43 in frontal cortex.**
BNN_20604	26	62	F	C9ORF72+ve	4		Yes
BNN_20993	55	43	M	C9ORF72+ve			
BNN_20613	89	50	M	C9ORF72+ve			Yes
BNN_30081	63	73	M	Negative	2	1	
BNN001.28407	93	67	F	Negative	1	2	
BNN001.30220	70	56	M	Negative			
BNN_29694	102	50	M	Negative	2		
BNN001.26126	119	71	M	Negative	1		
BNN001.26497	84	63	F	C9ORF72+ve			Yes
BNN001.28409	93	62	F	C9ORF72+ve	4	1	
BNN001.28792	51	68	M	C9ORF72+ve			
BNN_30175	84	69	F	Negative	4	4	Yes
BNN001.28790	105	70	M	Negative	2	2	
BNN001.26765	30	66	F	Negative	1	1	
BNN_22225	109	62	M	Negative			
BNN_18803	96	75	F	Negative			
BNN001.26729	131	83	F	Negative	1	1	
BNN001.31439	59	62	M	Negative	1		
Means	81	64	N/A	N/A	N/A	N/A	N/A
PMI = post-mortem interval; MRC = Medical Research Council.							

Supplementary Table 3 MRC numbers and conditions of controls.

Neurological healthy individuals					
**MRC numbers**	**PMI (hrs)**	**Age (yrs)**	**Sex**	**Braak stage**	**Thal stage**
BNN_19686	75	77	F	1	
BNN001.28960	126	37	F		
BNN001.28402	49	79	M	1	2
BNN001.28495	39	78	M	1	
BNN001.28959	86	39	M		
BNN001.29525	99	52	M		1
BNN001.28794	72	79	F	1	
BNN001.29526	67	47	M		
BNN001.28797	57	79	M		
BNN001.28406	72	79	M	2	2
BNN001.28793	72	79	F	2	1
Means	74	66	N/A	N/A	N/A
PMI = post-mortem interval; MRC = Medical Research Council.					

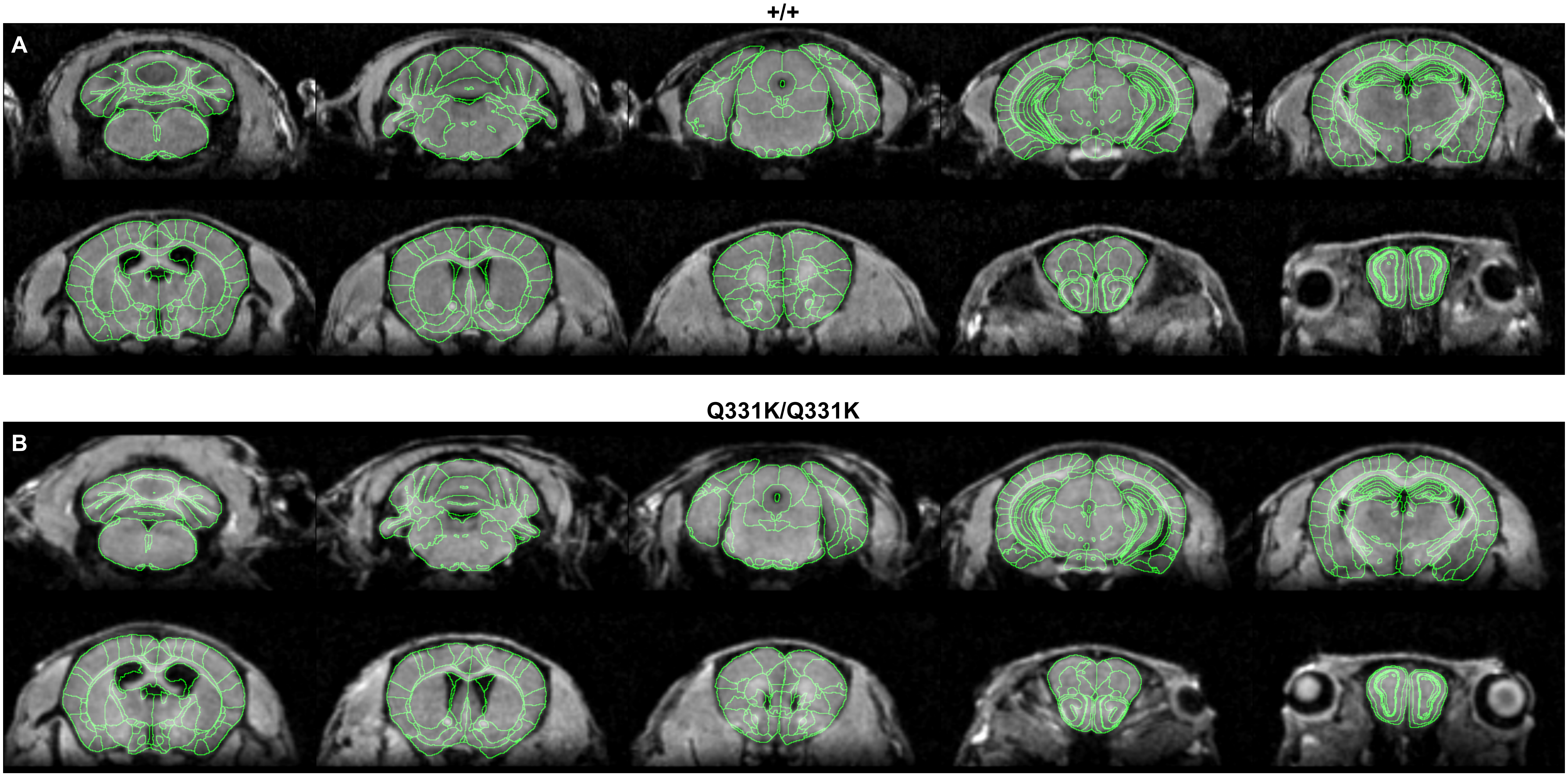

Supplementary Figure 1. Quality of MR image registration to the DSURQE mouse brain atlas.
Coronal slices of a WT (+/+) mouse (A) and a TDP43Q331K/Q331K (Q331K/Q331K) mouse (B). Green lines outline the DSURQE mouse brain atlas ROIs that have been mapped onto each mouse, illustrating the quality of the registration between the atlas and individual subjects. Arrow indicates misregistration of ventricles.

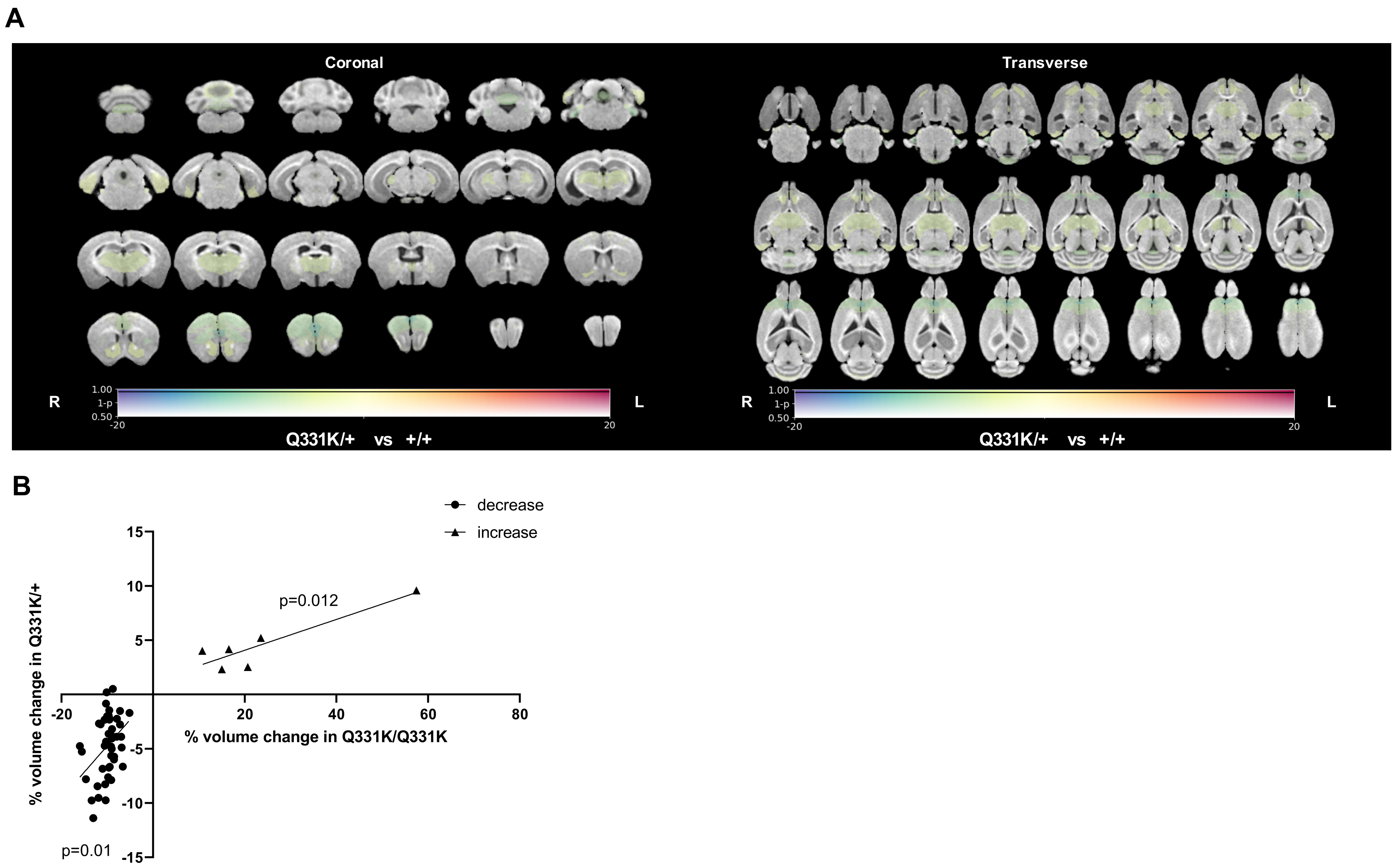

Supplementary Figure 2. No significant volume change in ROI analysis comparing heterozygous mutant to wild-type mice.
A) MRI study-specific template (coronal and transverse) with an overlay representing ROI volume differences at 7 months of age between WT and TDP-$43^{Q331K/+}$ mice. The colour of the overlay indicates the inter-group volume difference, while the transparency indicates the statistical significance. ROIs in which FWE-corrected P<0.05 are contoured in black. B) Correlation between % volume changes in ROIs in TDP-$43^{Q331K/+}$ and TDP-$43^{Q331K/Q331K}$ mice compared to WT mice.

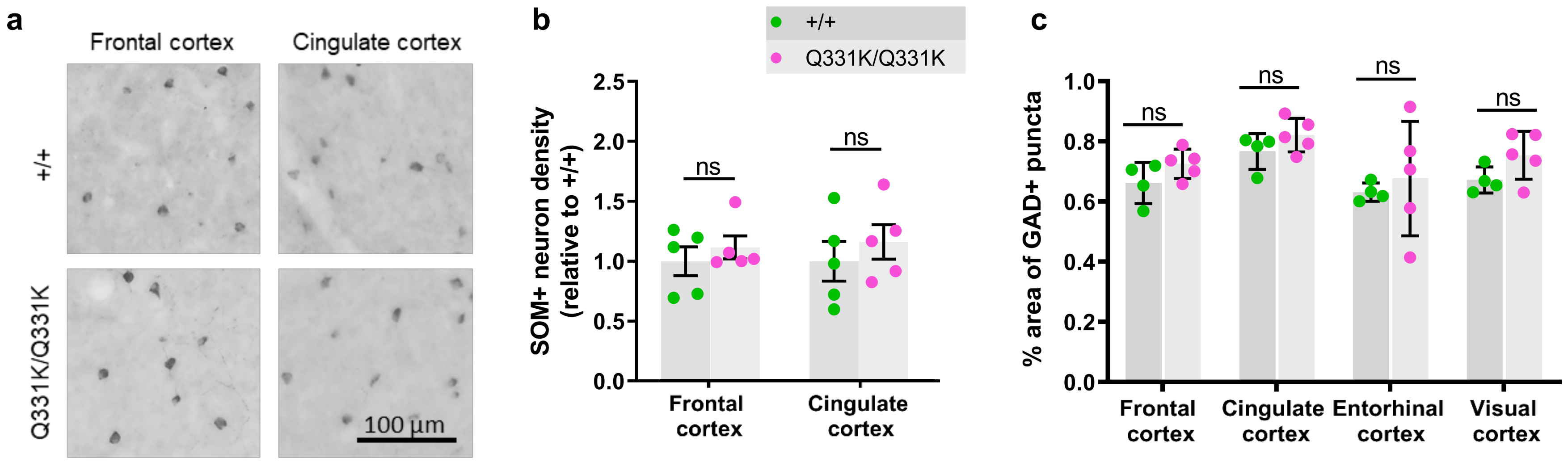

Supplementary Figure 3. No significant change in SOM+ interneuron density or GAD expression in mutant mice at 7 months of age. A) Representative images showing somatostatin positive (SOM+) neuron staining (dark brown) in frontal and cingulate cortices of 7-month old WT (+/+) and TDP43$^{Q331K/Q331K}$ (Q331K/Q331K) mice. B) Quantification of SOM+ neurons density in frontal cortex, ns P= 0.4713; cingulate cortex, ns P=0.4814 in 7m mutant mice compared to WT. Statistical analysis was performed by unpaired 2-tailed t-test (n=5/group). All data shown are mean ± s.e.m. C) Comparison of GAD67 immunostaining between mutant and wild-type mice in frontal cortex (p=0.1460), cingulate cortex (p=0.2026), entorhinal cortex (p=0.6569), and visual cortex (p=0.2026). Statistical analysis was performed by unpaired 2-tailed t-test (each dot represents one mouse). *ns* means not significant. All data shown are mean ± s.e.m.

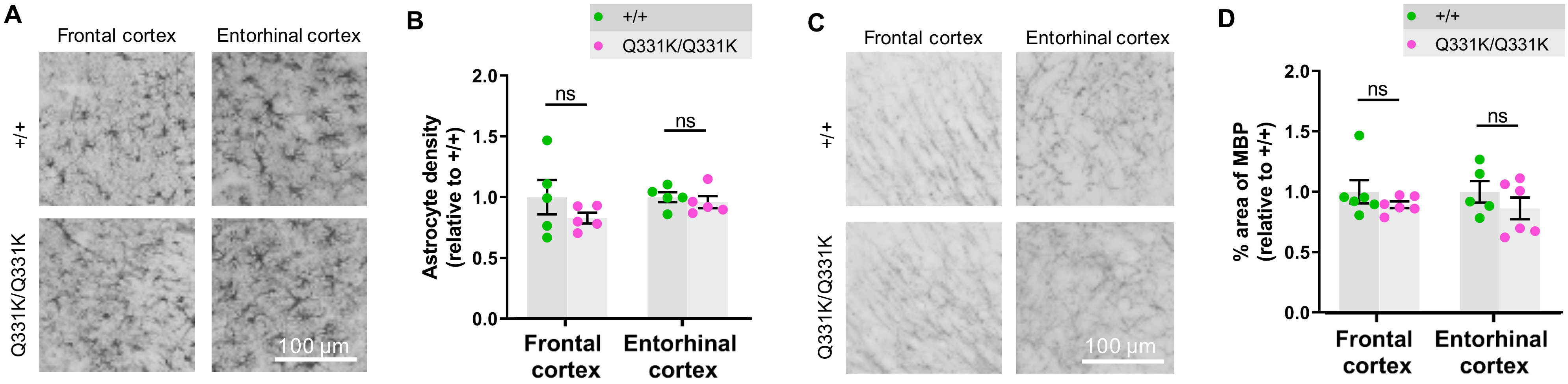

Supplementary Figure 4. No significant changes in astrocyte density and myelin basic protein % area in mutant mice.
A) Representative images showing astrocyte staining (dark brown) in given regions of 7-month old WT (+/+) and TDP43Q331K/Q331K (Q331K/Q331K) mice. B) Quantification of astrocyte density based on GFAP immunoreactivity comparing WT to mutant mice: frontal cortex, ns P= 0.2790; and entorhinal cortex, ns P=0.5450 . Statistical analysis was performed by unpaired 2-tailed t-test (n=5/group). All data shown are mean ± s.e.m. C) Representative images showing MBP staining (dark brown) in given regions of 7-month-old +/+ and Q331K/Q331K mice. D) Quantification of % area of MBP comparing WT to mutant mice: frontal cortex, ns P= 0.3064; entorhinal cortex, ns P=0.3123. Statistical analysis was performed by unpaired 2-tailed t-test (n=5-6/group). All data shown are mean ± s.e.m.

Supplementary Table 4. Genes differentially expressed in TDP-43^{Q331K} mice with roles in microglial functions.

Gene	Description	+/+	Q331K/Q331K	Fold Change	Microglial functions
**Microglia-specific in brain**					
20-month-old mice					
Tmem119	transmembrane protein 119	-	up	1.28	Microglia specific marker. Unknown function. (1)
**Expressed by microglia & other brain cells**					
5-month-old mice					
Cxcl5	chemokine (C-X-C motif) ligand 5	-	up	2.37	CXCL5 signalling is produced by activated microglia and is associated with BBB injury and white matter injury. (2)
Aif1l	allograft inflammatory factor 1-like	-	up	1.23	Alias IBA-2, 65% sequence homology with IBA-1.
Mertk	c-mer proto-oncogene tyrosine kinase	-	down	1.20	Induces phagocytosis signalling when activated. Involved in myelin phagocytosis.
Stat6	signal transducer and activator of transcription 6	-	down	1.28	Involved in microglia M2 phenotype induction (neuroprotective, anti-inflammatory). (3)
20-month-old mice					
C5ar1	complement component 5a receptor 1	-	up	1.42	Expressed by myeloid cells. Upregulation in the spinal cord of TDP43Q331K mice, increases in microglia. (4)
Cd55	CD55 antigen	-	up	1.24	Expressed by microglia in active lesions of myelin degradation in multiple sclerosis. (5)
Cd68	CD68 antigen	-	up	1.15	Up regulated in activated microglia.
Cd80	CD80 antigen	-	up	1.36	Expressed by activated microglia in vitro. Stimulates T-cells. (6)
Cd97	CD97 antigen	-	up	1.17	Expressed by microglia in active lesions of myelin degradation in multiple sclerosis. (5)
Csf1r	colony stimulating factor 1 receptor	-	up	1.17	Needed for microglial proliferation in the CNS. Its inhibition leads to microglial cell abolition in the CNS.
Cx3cr1	chemokine (C-X3-C) receptor 1	-	up	1.14	Expressed by microglia and macrophages. Its ligand, CXCL5 is expressed by neurons. Important for neuron-microglia communication.
Edn1	endothelin 1	-	up	1.29	Produced by activated microglia and astrocytes, implicated in initiating and sustaining reactive gliosis. (7)
Aif1	allograft inflammatory factor 1	-	up	1.16	Alias IBA-1.
Stat3	signal transducer and activator of transcription 3	-	up	1.07	Drives the expression of inflammatory genes in microglia. (8)
Mmd2	monocyte to macrophage differentiation-associated 2	-	up	1.14	High expression in the brain.
Siglece	sialic acid binding Ig-like lectin E	-	up	1.44	Myeloid lineage gene. Prevents neurotoxicity by binding to sialic acid present on the neuronal glycocalyx.
Siglech	sialic acid binding Ig-like lectin H	-	up	1.12	Myeloid lineage gene.

Chrna7	cholinergic receptor, nicotinic, α polypeptide 7	-	up	1.16	CHRNA7 activation leads to neuroprotective role of microglia. (9)
Sirt1	sirtuin 1	-	down	1.13	Proteine deacetylase, acts on histones and transcription factors. Activated by caloric restriction to inhinit inflammatory gene expression.
Tbk1	TANK-binding kinase 1	-	down	1.06	Haploinsufficiency causes ALS and FTD. (10)
**Not expressed by microglia, but influence microglia or have roles in inflammation**					
5-month-old mice					
C4a	complement component 4A	-	down	1.40	Role in microglia-mediated synaptic pruning. Increased activity associated with reduced synapse number. (11)
20-month-old mice					
C1qb	complement component 1, q subcomponent, β polypeptide	-	up	1.16	Tagging of synapses for removal by microglia. Upregulation in the spinal cord of TDP43 Q331K mice. (4)
Cd79b	CD79B antigen	-	up	2.23	Part of the B-cell antigen receptor complex. Related to BBB leakage. (12)
VCAM1	vascular cell adhesion molecule 1	-	up	1.23	Promotes adhesion of leukocytes to membranes, suggestive of BBB leakage and peripheral immune cell infiltration.

1. Bennett ML, Bennett FC, Liddelow SA, Ajami B, Zamanian JL, Fernhoff NB, et al. New tools for studying microglia in the mouse and human CNS. Proc Natl Acad Sci U S A. 2016;113(12):E1738-46.
2. Wang LY, Tu YF, Lin YC, Huang CC. CXCL5 signaling is a shared pathway of neuroinflammation and blood-brain barrier injury contributing to white matter injury in the immature brain. J Neuroinflammation. 2016;13:6.
3. Mou C, Liu B, Wang M, Jiang M, Han T. PGC-1-related coactivator (PRC) is an important regulator of microglia M2 polarization. J Mol Neurosci. 2015;55(1):69-75.
4. Lee JD, Levin SC, Willis EF, Li R, Woodruff TM, Noakes PG. Complement components are upregulated and correlate with disease progression in the TDP-43(Q331K) mouse model of amyotrophic lateral sclerosis. J Neuroinflammation. 2018;15(1):171.
5. Visser L, de Vos AF, Hamann J, Melief MJ, van Meurs M, van Lier RA, et al. Expression of the EGF-TM7 receptor CD97 and its ligand CD55 (DAF) in multiple sclerosis. J Neuroimmunol. 2002;132(1-2):156-63.
6. Satoh J, Lee YB, Kim SU. T-cell costimulatory molecules B7-1 (CD80) and B7-2 (CD86) are expressed in human microglia but not in astrocytes in culture. Brain Res. 1995;704(1):92-6.
7. D'Antoni S, Ranno E, Spatuzza M, Cavallaro S, Catania MV. Endothelin-1 Induces Degeneration of Cultured Motor Neurons Through a Mechanism Mediated by Nitric Oxide and PI3K/Akt Pathway. Neurotox Res. 2017;32(1):58-70.
8. Przanowski P, Dabrowski M, Ellert-Miklaszewska A, Kloss M, Mieczkowski J, Kaza B, et al. The signal transducers Stat1 and Stat3 and their novel target Jmjd3 drive the expression of inflammatory genes in microglia. J Mol Med (Berl). 2014;92(3):239-54.
9. Suzuki T, Hide I, Matsubara A, Hama C, Harada K, Miyano K, et al. Microglial alpha7 nicotinic acetylcholine receptors drive a phospholipase C/IP3 pathway and modulate the cell activation toward a neuroprotective role. J Neurosci Res. 2006;83(8):1461-70.
10. Freischmidt A, Wieland T, Richter B, Ruf W, Schaeffer V, Muller K, et al. Haploinsufficiency of TBK1 causes familial ALS and fronto-temporal dementia. Nat Neurosci. 2015;18(5):631-6.
11. Sekar A, Bialas AR, de Rivera H, Davis A, Hammond TR, Kamitaki N, et al. Schizophrenia risk from complex variation of complement component 4. Nature. 2016;530(7589):177-83.
12. UniProt. CD79B - B-cell antigen receptor complex-associated protein beta chain precursor - Homo sapiens (Human) - CD79B gene & protein [Available from: https://www.uniprot.org/uniprot/P40259.
